# Discovery and biosynthesis of bacterial drimane-type sesquiterpenoids from *Streptomyces clavuligerus*

**DOI:** 10.3762/bjoc.20.73

**Published:** 2024-04-16

**Authors:** Dongxu Zhang, Wenyu Du, Xingming Pan, Xiaoxu Lin, Fang-Ru Li, Qingling Wang, Qian Yang, Hui-Min Xu, Liao-Bin Dong

**Affiliations:** 1 State Key Laboratory of Natural Medicines, School of Traditional Chinese Pharmacy, China Pharmaceutical University, Nanjing 211198, China; 2 The Public Laboratory Platform, China Pharmaceutical University, Nanjing 211198, China

**Keywords:** bacterial terpenoid, cytochrome P450s, drimane-type sesquiterpenoid, *Streptomyces clavuligerus*, terpenoid biosynthesis

## Abstract

Drimane-type sesquiterpenoids (DMTs) are characterized by a distinctive 6/6 bicyclic skeleton comprising the A and B rings. While DMTs are commonly found in fungi and plants, their presence in bacteria has not been reported. Moreover, the biosynthetic pathways for DMTs have been primarily elucidated in fungi, with identified P450s only acting on the B ring. In this study, we isolated and characterized three bacterial DMTs, namely 3β-hydroxydrimenol (**2**), 2α-hydroxydrimenol (**3**), and 3-ketodrimenol (**4**), from *Streptomyces clavuligerus*. Through genome mining and heterologous expression, we identified a *cav* biosynthetic gene cluster responsible for the biosynthesis of DMTs **2**–**4**, along with a P450, CavA, responsible for introducing the C-2 and C-3 hydroxy groups. Furthermore, the substrate scope of CavA revealed its ability to hydroxylate drimenol analogs. This discovery not only broadens the known chemical diversity of DMTs from bacteria, but also provides new insights into DMT biosynthesis in bacteria.

## Introduction

**Figure 1:**
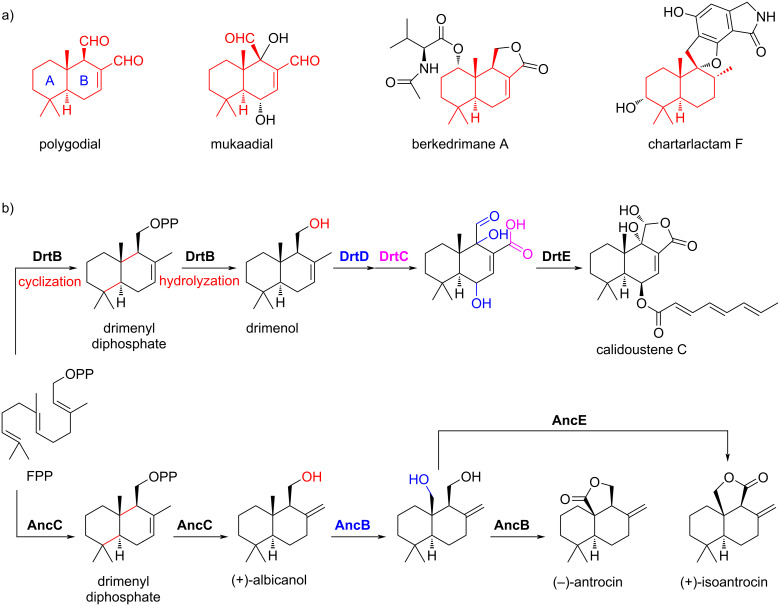
(a) Representative bioactive drimane-type sesquiterpenoids (DMTs). (b) Reported biosynthetic pathways of DMTs from fungi.

Terpenoids, encompassing over 11,000 compounds (http://dnp.chemnbase.com), are the most diverse group of natural products found in nature [1]. All terpenoids are biosynthesized from C_5_ carbon units, which are sourced from the isoprenoid building blocks isopentenyl diphosphate (IPP) and dimethylallyl diphosphate (DMAPP) from the mevalonate (MVA) and 2-*C*-methyl-ᴅ-erythritol 4-phosphate (MEP) pathways [2–3]. Sesquiterpenoids, synthesized from farnesyl diphosphate (FPP) which is composed of three C_5_ units, hold significant biological and industrial value, particularly in the realm of perfumery [4]. Among these, drimane-type sesquiterpenoids (DMTs) are distinct due to their chemical structures, which feature a decahydronaphthalene core adorned with methyl groups, mirroring the A/B rings found in labdane-derived diterpenoids [5–6] (Figure 1a). DMTs exhibit significant biological activities, such as those found in polygodial, which serves as an antifeedant for various herbivorous insects [7]. Additionally, berkedrimane A, mukaadial, and chartarlactam F demonstrate caspase-3 inhibition, antifungal, and antihyperlipidemic activities, respectively [6,8–12]. Collectively, these examples emphasize the potential of DMTs as substances with broad and significant biological activities.

The biosynthetic pathways for DMTs, especially for calidoustene C, (+)-isoantrocin, and (−)-antrocin, have been extensively elucidated [6,13–20] (Figure 1b). In the biosynthesis of calidoustene C, DrtB from *Aspergillus calidoustus* functions as a dual-functional enzyme, comprising two domains: a HAD-like hydrolase domain fused with a terpene cyclase domain. Initially, FPP is cyclized into the drimenyl diphosphate in a class II terpene cyclase manner, which is then processed by the hydrolase domain to form drimenol [21]. Subsequently, this molecule undergoes further modifications by two cytochrome P450s monooxygenases (P450s, DrtC and DrtD) and one acyltransferase (DrtE) to produce calidoustene C [13]. This cyclization is similar with the course of AncC from *Antrodia cinnamomea*, which performs a similar role to DrtB, engaging two P450s (AncE and AncB) to synthesize (+)-isoantrocin and (−)-antrocin [18]. Notably, the P450s identified in fungi and plants predominantly modify the B-ring of DMTs [15–16,18].

**Figure 2:**
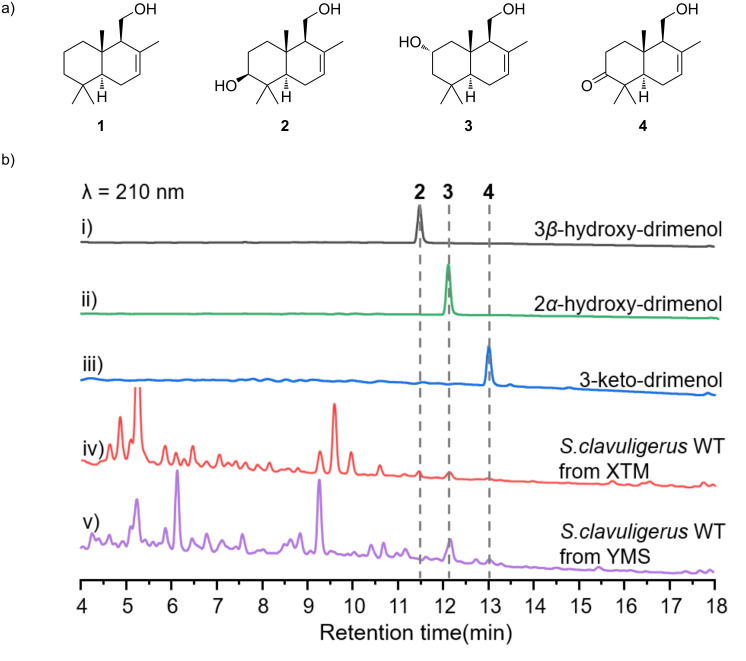
Chemical structures and HPLC analysis. (a) Chemical structures of isolated sesquiterpenes **2**–**4** from *S. clavuligerus*. (b) HPLC chromatograms comparing standards with metabolites extracted from *S. clavuligerus* cultured in YMS and XTM media.

DMTs are commonly found in plants and fungi [6,9,13,15]. While enzymes associated with DMT biosynthesis have been identified in bacteria, the corresponding natural DMTs have not been discovered [17]. In this study, we isolated and characterized three drimenol congeners (**2**–**4**) from *Streptomyces clavuligerus* (Figure 2a). In the genome of *S. clavuligerus*, we identified a *cav* biosynthetic gene cluster (BGC) that typically includes three P450s, one class II drimenyl diphosphate synthases (DMS), and one characteristic Nudix hydrolase responsible for the DMTs biosynthesis. Among the three P450s, CavA was the only oxygenase acting on drimenol and versatile in oxidizing C-2 and C-3 positions of the A ring of drimenol by heterologous expression and gene knockout. Furthermore, we tested the substrate promiscuity of CavA with drimenol analogs. CavA exhibited the ability to accept albicanol (**5**) and drim-8-ene-11-ol (**6**) as substrates, leading to the formation of the hydroxylated derivatives **7**–**9**. This study marks the first discovery of natural DMTs from bacteria and unveils the role of CavA in a novel late-stage modification pathway, expanding DMT biosynthesis beyond fungi and plants.

## Results and Discussion

### Discovery of three DMTs in *S. clavuligerus*

*S. clavuligerus* is notably significant in industrial applications, renowned for its production of diverse natural products with chemical structures and bioactivities, such as cephamycin C, clavulanic acid, and isopenicillin N [22–24]. Genomic sequencing of *S. clavuligerus* has revealed 48 potential secondary metabolite BGCs, and our analysis indicated that 16 of them are potential terpene-producing BGCs, indicative of its extensive capacity for diverse metabolite production [25]. In our large-scale fermentation of *S. clavuligerus*, aimed at uncovering novel bacterial terpenoids, we observed that specific culture conditions are crucial for metabolite production. Different culture media can lead to the biosynthesis of varied compounds. Specifically, in this study, *S. clavuligerus* was cultivated in two terpene favored media, XTM and YMS [26–27], resulting in three drimenol (**1**) congeners from the extracts: 3β-hydroxydrimenol (**2**), 2α-hydroxydrimenol (**3**), and 3-ketodrimenol (**4**) (Figure 2a). HPLC analysis of metabolites from different culture media showed that YMS medium was more conducive to produce compound **3** (Figure 2b and Table S1 in Supporting Information File 1).

The chemical structures of these isolated compounds were mainly elucidated through comparative ^1^H and ^13^C NMR spectra and by comparing analytical data with existing literature reports [28–29] (see Supporting Information File 1, Figures S1–S7). The ^1^H NMR spectrum of compound **2** exhibited characteristic signals for one olefinic proton at δ_H_ (5.48) and four methyl groups at δ_H_ (0.83, 0.87, 0.98 and 1.77). Its ^13^C and DEPT NMR spectra showed 15 carbon resonances, including four methyl groups, four methylenes, four methines, and three quaternary carbon atoms. This information suggests that it may be a drimenol congener and the structure was further supported by comparison of analytical data with a literature report [29]. The ^1^H NMR spectrum of compound **3** is similar to that of **2**, showing four methyl groups and one olefinic methyl singlet, indicating structural resemblances with minor variations. It was ultimately determined through literature comparison that the only difference between compounds **3** and **2** is the position of the hydroxy group [28]. Analyses of the NMR data of compound **4** concluded that it is an analogue of **2**. In comparison with **2**, compound **4** has a ketone carbonyl signal at δ_C_ 219.1 and we finally confirmed its structure by comparing it with literature data [29]. This marks the first discovery of natural DMTs from bacteria, surpassing previous findings of bacterial DMSs which focused only on the enzyme itself without reporting natural DMT or exploring associated BGCs [17]. Additionally, while drimentines, bacterial meroterpenoids, share the drimanyl scaffold, their biosynthetic pathways, particularly the terpene cyclases synthesizing the drimanyl structures, show substantial divergence from DMT pathways [21,30–32]. This fundamental difference in biosynthetic mechanisms serves to categorize DMSs and drimentines into distinct subfamilies within the sesquiterpenoids. Our discovery significantly broadens the scope for future exploration of bacterial DMTs.

### Discovery and identification of a DMS in the *cav* BGC in *S. clavuligerus*

**Figure 3:**
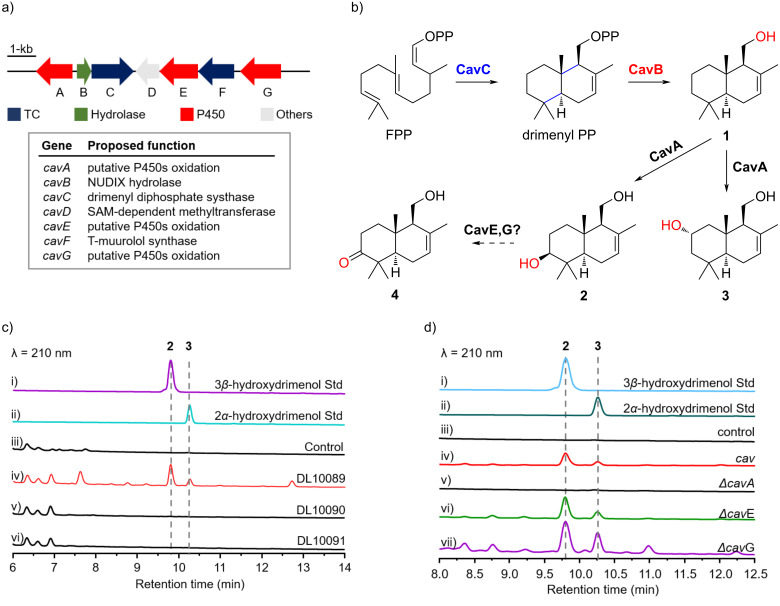
Biosynthesis of drimenol congeners. (a) The *cav* BGC. (b) Proposed biosynthetic pathway for drimenol congeners **2**–**4**. (c) HPLC analysis of metabolites from genetically engineered *Streptomyces* strains, with *S. avermitilis* SUKA22 harboring the empty pSET152 vector as the control. (d) HPLC analysis of metabolites co-expressed with drimenyl diphosphate synthases (CavC), Nudix hydrolase (CavB), and one of the P450s, either CavA (DL10089), CavE (DL10090), or CavG (DL10091).

To explore the BGC responsible for DMTs production in *S. clavuligerus*, we performed bioinformatics research utilizing the EFI-genome neighborhood tool (EFI-GNT), sequence alignment, and manual BLAST analysis [33]. Leveraging our previous discovery of SsDMS from *Streptomyces showdoensis*, we identified a homologous terpene cyclase (sclav_p0068) in *S. clavuligerus*. This enzyme shares a 50% sequence similarity with SsDMS, including a conserved “DXDD” motif, indicating its potential as a class II sesquiterpene synthase (Figure S8, Supporting Information File 1) [20]. Further analysis of adjacent genes revealed a Nudix hydrolase located upstream of the putative DMS, similar to the reported situation with SsDMS [19]. Inspection of the BGC identified additional three P450s. Thus, the entire cluster mainly encompasses a DMS (CavC), a Nudix hydrolase (CavB), a class I terpene cyclase (CavF), and three P450s (CavA, CavE, and CavG), and it was designated as the *cav* cluster (Figure 3a and Table S2 in Supporting Information File 1).

To validate the function of the presumed DMS, we cloned its gene into the pETDuet-1 vector to form plasmid pLD10050, which was transformed into our truncated artificial FPP-overproduction system in *E. coli* to yield strain DL10092 [34]. Analysis of the fermentation extracts revealed that DL10092 displayed a new peak in its HPLC profile compared to the control of wild-type *E. coli* BL21 (DE3) (see Figure S9 in Supporting Information File 1). The fermented product appeared as an alcohol, likely due to the activity of the endogenous hydrolase in *E. coli*. The retention time of the new peak was identical with the drimenol standard, suggesting successful synthesis of drimenol. This confirms CavC as a DMS, and the *cav* BGC is likely responsible for the biosynthesis of compounds **2**–**4**.

### CavA, a P450, responsible for the formation of C-2 and C-3 hydroxy groups

It was hypothesized that compound **1** serves as a precursor for derivatives **2**–**4**. The biosynthetic pathway is likely to involve enzymatic modifications at the C-2 and C-3 positions, leading to the formation of diols **2** and **3**, with additional oxidation steps producing ketone **4** (Figure 3b). This supports the presence of three P450s in the *cav* cluster. We initially cloned and expressed the complete *cav* BGC in three model *Streptomyces* species (Tables S3–S5 in Supporting Information File 1). Notably, *Streptomyces lividans* TK64 DL10081, containing the entire *cav* BGC, produced only compound **2** in three media (PTMM, XTM, and ISM3; Figure S10 in Supporting Information File 1). Interestingly, *S. avermitilis* SUKA22 DL10085 can produce **2** and **3** in XTM medium (Figure 3c). The subsequent 7.5 L fermentation of strain DL10085 in XTM medium resulted in a significant production of compounds **2** (14 mg) and **3** (5 mg), highlighting the crucial role of P450s in the biosynthesis. However, compound **4** was not detected in the heterologous expression products, potentially due to the involvement of a dehydrogenase outside the *cav* BGC.

An in-depth study of the specific roles of the P450 genes was conducted by creating single-gene deletion mutants from strain *S. avermitilis* SUKA22 DL10085. The DL10086 strain, with the deletion of the *cavA* gene, failed to produce **2** or **3**, suggesting the vital role of *cavA* in *cav* BGC. To further substantiate the hypothesis, *cavB* and *cavC* were co-expressed with each of the three P450s in *S. avermitilis* SUKA22, creating three recombinant strains (DL10089, DL10090, and DL10091; Tables S3–S5 in Supporting Information File 1). Notably, the DL10089 strain, co-expressing drimenol synthase and CavA, successfully produced **2** and **3**. This result not only corroborates our initial findings but also confirms the CavA role in hydroxylating the C-2 and C-3 positions of drimenol (Figure 3d). However, the remaining two P450 enzymes, CavE and CavG, appear to be non-functional either in the native *S. clavuligerus* or heterologous expression systems. Given the detection of two terpene cyclases (CavC and CavF) and the exclusive DMTs generated, we also speculated that the characterized *cav* BGC may include two separate BGCs situated closely together. Future efforts will be focused on uncovering the roles of the other three enzymes (CavF, CavE, and CavG).

### Substrate scope of CavA with drimenol analogs

**Figure 4:**
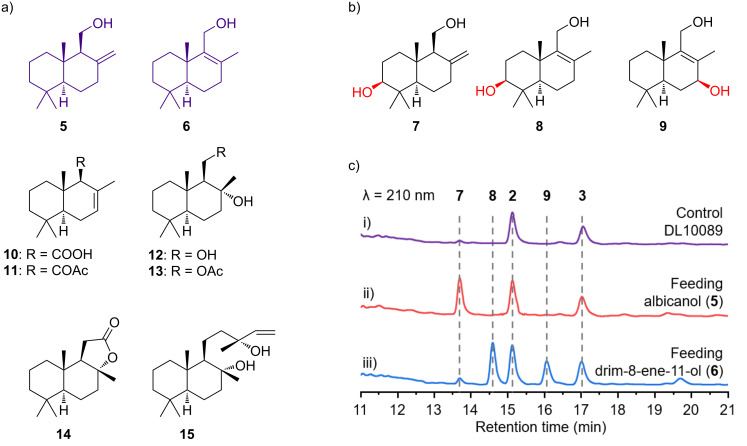
Substrate scope of CavA. (a) The chemical structures of eight natural products that were used for feeding experiments. (b) The three hydroxylated products generated from feeding experiments. (c) HPLC analysis of metabolites from *S. avermitilis* SUKA22 strain DL10089 cultured in XTM medium with albicanol (**5**) and drim-8-ene-11-ol (**6**) supplementation.

P450s are renowned for their remarkable versatility as biocatalysts, capable of catalyzing a wide range of reactions and processing a variety of substrates [35–36]. They effectively recognize different structural types, including aromatic compounds, polyketides, terpenes, peptides, and carbohydrates [37–40]. To investigate the substrate spectrum of CavA more closely, eight compounds were chosen for this study (Figure 4a). Due to the insolubility of CavA in *E. coli*, the study employed in vivo substrate screening. The *S. avermitilis* SUKA22 DL10089 was cultivated in XTM medium with the supplement of various substrates for five days. HPLC analysis indicated that CavA specifically oxidizes albicanol (**5**) and drim-8-ene-11-ol (**6**) to synthesize **7**–**9**, while other substrates showed no reactivity with CavA (Figure 4c). Following this, we increased the substrate concentration to enrich and isolate these products, elucidating their structures through NMR analysis (Figure 4b). In these experiments, CavA was found to hydroxylate drim-8-ene-11-ol (**6**) at the C-3 and C-7 positions to produce **8** and **9**, respectively, while albicanol (**5**) was modified only at the C-3 position. Compound **7** is a known natural product, and its chemical structure was determined by comparing the ^1^H and ^13^C NMR data with the literature (Figures S11 and S12 in Supporting Information File 1) [41]. Although the chemical structure of compound **8** has been documented, its ^1^H and ^13^C NMR data were not fully reported [42]. Therefore, we conducted comprehensive 1D and 2D NMR experiments on it (Figures S13–S18, Supporting Information File 1) and summarized the resulting data in Table S6 (Supporting Information File 1). For compound **9**, despite sharing an identical planar structure with diaporol E, the configurations of the C7 hydroxy groups are opposite, as evidenced by significant chemical shift differences in ^13^C NMR (Δδ_C_ = 3.3) (Figures S19–S25, Supporting Information File 1) [43]. Moreover, the key correlation observed between H5 and H7 in the ROESY spectrum provides additional support for the β-configuration of this hydroxy group (Figure S26 and Table S7 in Supporting Information File 1). These findings from both the heterologous expression and substrate spectrum analysis indicate the particular affinity of CavA for the C-3 position, which might be used for chemoenzymatic synthesis of C-3 hydroxylated drimanyl meroterpenoids using drimenol and its analogues as substrates. However, it is important to note that CavA exhibits limited substrate promiscuity, predominantly targeting the drimenol skeleton with minor variations. This selectivity may be attributed to the structural configuration of the enzyme, which appears to be finely tuned to recognize and interact with specific features of the drimenol framework.

## Conclusion

In this study, we isolated and characterized three bacterial DMTs (**2**–**4**) derived from *S. clavuligerus*. This finding challenges the traditional focus on extracting DMTs exclusively from fungi and plants, establishing a new avenue for the discovery of DMTs. Subsequently, we identified the *cav* BGC responsible for the production of compounds **2** and **3** and elucidated its biosynthetic pathway by heterologous expression. Specifically, our study unveils a P450, CavA, capable of oxidizing the C-2 and C-3 positions of drimenol, representing the first native P450 that activates the A-ring of DMTs. The in vivo experiments further expanded the substrate scope of CavA to include albicanol (**5**) and drim-8-ene-11-ol (**6**), showcasing the enzyme's biocatalytic potential. This study establishes a foundation for biocatalysts targeting the A-ring of drimenol, which might be beneficial for the efficient chemoenzymatic synthesis of DMTs or drimanyl indoles [44].

## Supporting Information

File 1 Experimental part and supplementary figures and tables.

## Data Availability

All data that supports the findings of this study is available in the published article and/or the supporting information to this article.
