# Supplementary material for: Discovery and biosynthesis of bacterial drimane-type sesquiterpenoids from Streptomyces clavuligerus
Source: Beilstein J Org Chem. 2024 Apr 16;20:815–22. doi: 10.3762/bjoc.20.73 (PMC11035983; doi:10.3762/bjoc.20.73)
Supplement: File 1 — Experimental part and supplementary figures and tables. [file Beilstein_J_Org_Chem-20-815-s001.pdf]

BEILSTEIN JOURNAL OF ORGANIC CHEMISTRY

Supporting Information

for

Discovery and biosynthesis of bacterial drimane-type sesquiterpenoids from *Streptomyces clavuligerus*

Dongxu Zhang, Wenyu Du, Xingming Pan, Xiaoxu Lin, Fang-Ru Li, Qingling Wang, Qian Yang, Hui-Min Xu and Liao-Bin Dong

Beilstein J. Org. Chem. **2024,** *20,* 815–822. doi:10.3762/bjoc.20.73

Experimental part and supplementary figures and tables

License and Terms: This is a supporting information file under the terms of the Creative Commons Attribution License (https://creativecommons.org/licenses/by/4.0). Please note that the reuse, redistribution and reproduction in particular requires that the author(s) and source are credited and that individual graphics may be subject to special legal provisions.
The license is subject to the *Beilstein Journal of Organic Chemistry* terms and conditions: (https://www.beilstein-journals.org/bjoc/terms)

Table of contents

Supplementary methods S2–S8

Supplementary Tables S9–S14

Supplementary Figures S15-S30

Supplementary References S31–S32

Materials and methods

General experimental procedures.

Restriction enzymes were purchased from Takara (Beijing, China). Primers were synthesized by Sangon Biotech (Shanghai, China). Gene sequencing was performed by Sangon Biotech (Shanghai, China). Phanta Super-Fidelity DNA Polymerase, ClonExpress II One Step Cloning Kit, and DNA gel extraction were used to clone DNA into plasmid, which was purchased from Vazyme Biotech (Nanjing, China). All plasmids were extracted from *Escherichia coli* DH5α strains with plasmid extraction kits (Vazyme Biotech, Nanjing, China). Other chemicals, biochemical, and media components were purchased from standard commercial sources. The ^{1}H and ^{13}C NMR experiments were conducted using a Bruker Avance NEO spectrometer with a 400 MHz operating frequency for ^{1}H nuclei and a 100 MHz operating frequency for ^{13}C nuclei. HPLC analysis performed on an Agilent 1260 Infinity with an Agilent Poroshell 120 EC-C18 column. Preparative HPLC was performed on an Agilent 1260 Prep Infinity LC with a VWD detector equipped with an Agilent Eclipse XDB-C18 column (250 mm × 21.2 mm, 7 μm). MPLC was performed on a Sanotac Purifier-100 with ODS from Fuji Silysia Chemical. Optical rotations were obtained using an AUTOPOL IV automatic polarimeter (Rudolph Research Analytical). HRESIMS data were obtained using an Agilent G6230 Q-TOF mass instrument.

DNA manipulation and plasmid construction.

The genomic sequence of *Streptomyces clavuligerus* CGMCC 4.5336 was analyzed using

enzyme function initiative enzyme similarity tool (EFI-EST), antiSMASH, and NCBI to identify biosynthetic gene clusters (BGC) [1,2]. The *S. clavuligerus* genomic DNA was extracted using the DC103 kit. The target BGC was divided into three fragments and amplified from the genomic DNA using three pairs of primers listed in Table S4 and Phanta Max Super-Fidelity DNA Polymerase. The PCR products were purified and treated with ClonExpress Mix, and then cloned into plasmid pSET152-ermE between EcoRV and BamHI sites by homologous recombination to afford pLD10101, which has apramycin resistance gene. Similarly, the three P450 genes were amplified using corresponding primers by PCR, and then cloned into the EcoRV and BamHI sites of the pSET152-ermE vector, along with the amplified drimenol synthase and Nudix hydrolase. Using pLD10101 as a template, the three P450 genes were individually knocked out, resulting in four plasmids. All plasmids were verified by DNA sequencing. These plasmids were transferred into the *E. coli* ET12567/pUZ8002 strain individually, and further introduced into model *Streptomyces* by conjugation.

Culture conditions

E. coli strains carrying the plasmids were cultured in lysogeny broth (LB) medium and screened by apramycin. *S. clavuligerus* was cultivated at 28 °C on solid ISP2 medium, while other *Streptomyces* strains were cultured at 28 °C on solid MS or ISP2 media. The *E. coli–Streptomyces* mixtures were carefully plated onto ISP4 solid medium supplemented with 20 mM $MgCl_2$ and incubated overnight at 28 °C. Three fermentation media (PTMM,

XTM, and ISM3, specific composition of each medium is listed below) were used to ferment recombinant *Streptomyces* strains [3-5]. Wild-type strains were cultured in PTMM, XTM, and YMS media. Fresh spores from each recombinant strain were inoculated into 50 mL Tryptone Soy Broth (TSB) supplemented with apramycin in 250 mL baffled flasks. The TSB liquid cultures were incubated in 28 °C shaker with 250 rpm for 48 h. Then, 4% (v/v) seed culture was transferred to 2.5-L baffled Erlenmeyer flasks containing 500 mL fermentation medium, and grown in 28 °C shaker with 250 rpm for 7 days. After fermentation, the culture was extracted with EtOAc directly, and EtOAc was removed under vacuum to obtain crude extract which can be used for terpenoids isolation.

ISP2 medium (1 liter): 4 g yeast extract, 10 g malt extract, 4 g dextrose, and 20 g agar, pH 7.3

MS medium (1 liter): 20 g ᴅ-mannitol, 20 g soya flour, 3 g $CaCO_3$, and 20 g agar, pH 7.2

ISP4 medium (1 liter): 10 g soluble starch, 1 g K_2HPO_4, 1 g $MgSO_4$, 1 g NaCl, 2 g $(NH_4)_2SO_4$, 2 g $CaCO_3$, 1 mg $FeSO_4$, 1 mg $MnCl_2$, 1 mg $ZnSO_4$, 20 g agar, pH 7.2

PTMM medium (1 liter): 40 g dextrin, 40 g lactose, 5 g yeast extract, 5 g MOPS, and 10 mL trace elements ($ZnCl_2$ 40 mg/L, $FeCl_3{\cdot}6H_2O$ 200 mg/L, $CuCl_2$ 10 mg/L, $MnCl_2{\cdot}4H_2O$ 10 mg/L, $Na_2B_4O_7{\cdot}10H_2O$ 10 mg/L, and $(NH_4)_6Mo_7O_{24}{\cdot}4H_2O$ 10 mg/L), pH 7.3

XTM medium (1 liter): 10 g yeast extract, 10 g malt extract, 10 g maltose, and 10 mL trace elements ($ZnCl_2$ 40 mg/L, $FeCl_3{\cdot}6H_2O$ 200 mg/L, $CuCl_2$ 10 mg/L, $MnCl_2{\cdot}4H_2O$ 10 mg/L, $Na_2B_4O_7{\cdot}10H_2O$ 10 mg/L, and $(NH_4)_6Mo_7O_{24}{\cdot}4H_2O$ 10 mg/L), pH 7.2

ISM3 medium (1 liter): 15 g yeast extract, 10 g malt extract, 0.5 g $MgSO_4$, 0.3 g $FeCl_3 \cdot 6H_2O$, and 20 g glucose, pH 7.0

YMS medium (1 liter): 4 g yeast extract, 10 g malt extract, 4 g soluble starch, pH 7.0

Heterologous expression, extraction, and HPLC analysis.

Plasmid pLD10101 was transferred into the *E. coli* ET12567/pUZ8002 strain, and further introduced into three *Streptomyces* strains (*S. lividans* TK64、*S. coelicolor* M1154, and *S. avermitilis* SUKA22) by conjugation. After 20 h incubation at 28 °C, replate nalidixic acid and apramycin on ISP4 solid media to select for successful *Streptomyces* exconjugants. Identify positive colonies were screened through PCR with corresponding primers (Table S4). Other plasmids were individually transferred into *S. avermitilis* SUKA22 to obtain recombinant *Streptomyces* strains. All recombinant *Streptomyces* strains were fermented in three different media (PTMM, XTM and ISM3) in 28 °C shaker with 250 rpm for 7 days. (Feeding experiment: After one day of inoculating the seed culture, the substrate dissolved in DMSO is fed.) The 50 mL fermentations were transferred to 50 mL conical tubes and centrifuged at 3750 rpm for 20 min to separate broth and mycelium. Soak the mycelium in acetone, remove the solvent, and then extract the remaining solution and the broth with ethyl acetate (EtOAc). Then dissolve the broth and mycelium extract in CH_3OH, filter it through a 0.22 μm filter, and proceed with HPLC analysis. The solvent gradient for the HPLC analysis is listed in Table S1.

Isolation and structural identification

S. clavuligerus was fermented individually on a 15 L scale using both the YMS and XTM media and *S. avermitilis* SUKA22 DL10085 was fermented in XTM medium with a 7.5 L scale. After 7 days of fermentation, the culture was centrifuged at 3750 rpm for 20 min to separate broth and mycelium. Combine the supernatant of YMS and XTM fermentation of *S. clavuligerus*, then the broth was extracted three times with EtOAc. Soak the mycelium in acetone, remove the solvent, and then extract the remaining solution with EtOAc. After vacuum concentration, combine the extraction of broth and mycelium. The XTM fermentation of *S. avermitilis* SUKA22 DL10085 was also processed using the same method. Crude extracts were fractionated by medium pressure liquid chromatography (MPLC) over ODS column eluted with a linear gradient CH_3OH–H_2O system from 20% CH_3OH to 100% CH_3OH. The target fractions were further purified by semi-preparative HPLC. Finally, the target components were subjected to ^{1}H and ^{13}C NMR experiments to determine their chemical structures, which were then compared to reported data [7-9].

3β-Hydroxydrimenol (2). White, amorphous solid. ^{1}H NMR (400 MHz, CD_3OD): δ_H = 5.46 (s, 1H), 3.78 (dd, *J* = 11.1, 3.1 Hz, 1H), 3.57 (dd, *J* = 11.1, 6.5 Hz, 1H), 3.19 (dd, *J* = 11.0, 4.8 Hz, 1H), 2.05 (dt, *J* = 13.3, 3.5 Hz, 1H), 1.98 (s, 2H), 1.81 (br, 1H), 1.76 (s, 3H), 1.63 (s, 2H), 1.28 (s, 1H), 1.17–1.21 (m, 1H), 0.96 (s, 3H), 0.85 (s, 3H), 0.81 (s, 3H) ppm; ^{13}C NMR (100 MHz, CD_3OD) δ_C = 134.9, 123.7, 79.7, 61.1, 58.2, 51.0, 39.7, 38.9, 36.8, 28.7, 28.1, 24.4, 22.1, 15.9, 14.9 ppm.

2α-Hydroxydrimenol (3). Colorless oil. ^{1}H NMR (400 MHz, $CDCl_3$): δ_H = 5.55 (m, 1H), 3.96–3.83 (m, 2H), 3.78 (dd, J = 11.4, 4.9 Hz, 1H), 2.30 (dt, J = 12.0, 3.2, 1H), 2.08–1.96 (m, 1H), 1.92 (m, 1H), 1.86 (m, 1H), 1.79 (m, 3H), 1.76 (m, 1H), 1.17 (dd, J = 12.0, 4.8 Hz, 2H), 1.04 (m, 1H), 0.94 (s, 6H), 0.91 (s, 3H) ppm; ^{13}C NMR(100 MHz, $CDCl_3$): δ_C = 132.6, 124.0, 65.0, 60.8, 57.1, 51.0, 49.2, 49.1, 37.9, 34.6, 33.3, 23.4,23.0, 21.8, 15.8 ppm.

3-Ketodrimenol (4). Colorless oil. ^{1}H NMR (400 MHz, CD_3OD): δ_H = 5.51 (s, 1H), 3.82 (dd, J = 11.3, 3.8 Hz, 1H), 3.67 (dd, J = 11.3, 6.0 Hz, 1H), 2.82 (td, J = 14.5, 5.3 Hz, 1H), 2.37 (ddd, J = 13.4, 5.3, 3.6 Hz, 1H), 2.21 (dt, J = 14.5, 3.8 Hz, 1H), 2.10 (m, 1H), 1.94 (m, 2H), 1.77 (m, 3H), 1.61 (m, 2H), 1.11 (s, 3H), 1.07 (s, 3H), 1.04 (s, 3H) ppm; ^{13}C NMR (100 MHz, CD_3OD): δ_C = 219.1, 135.1, 123.5, 61.0, 57.2, 52.8, 48.6, 39.4, 36.8, 35.5, 25.7, 24.9, 22.7, 22.1, 14.4 ppm.

3β-Hydroxy-albicanol (7). White, amorphous solid. ^{1}H NMR (400 MHz, $CDCl_3$): δ_H =4.96(s, 1H), 4.66(s, 1H), 3.85–3.72 (m, 2H), 3.27 (dd, J = 11.8, 4.1 Hz, 1H), 2.44 (ddd, J = 12.9, 4.4, 2.4 Hz, 1H), 2.08–1.89 (m, 3H), 1.76 (ddt, J = 13.9, 6.1, 3.0 Hz, 1H), 1.73–1.66 (m, 2H), 1.66–1.51 (m, 1H), 1.49–1.30 (m, 3H), 1.12 (dd, J = 12.5, 2.8 Hz, 1H), 1.00 (s, 3H), 0.77 (s, 3H), 0.72 (s, 3H); ^{13}C NMR(100 MHz, $CDCl_3$): δ_C =147.4, 106.8, 78.7, 58.9, 58.8, 54.5, 39.2, 38.8, 37.8, 37.1, 28.4, 27.8, 23.9, 15.6, 15.4 ppm.

3β-Hydroxy-drim-8-ene-11-ol (8). White, amorphous solid. ^{1}H and ^{13}C NMR data see Table S6.

7β-Hydroxy-drim-8-ene-11-ol (9). White, amorphous solid. $[\alpha]_{20}^{D}$ +21.0 (c 0.1, MeOH). ^{1}H and ^{13}C NMR data see Table S7. (–)-HRESIMS *m/z* 283.1916 $[M + HCOO]^{-}$ (calcd for $C_{16}H_{27}O_4$, 283.1909).

Table S1: HPLC analysis of fermentation samples.

Time/min	H_2O	CH_3CN (containing 0.1% formic acid)
0	90%	10%
22	0%	100%
25	0%	100%
25.01	90%	10%
28	90%	10%

Table S2: Predicted gene functions and BLAST results of *cav* BGC.

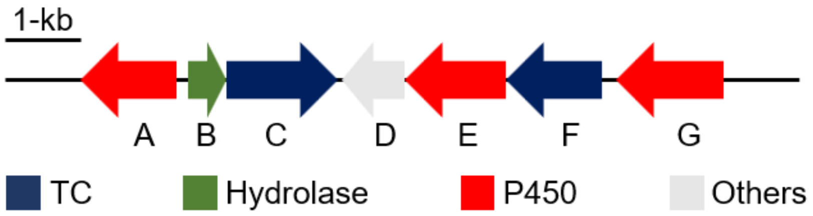

Protien	Accession numbers	Size(aa)	Proposed function
CavA	WP_003956085	423	cytochrome P450
CavB	WP_003956086	165	NUDIX hydrolase
CavC	WP_009998890	488	Terpenoid cyclase
CavD	WP_003956088	275	SAM-dependent methyltransferase
CavE	EDY50540	445	cytochrome P450
CavF	WP_003956090	418	Terpenoid cyclase
CavG	WP_009998888	471	cytochrome P450

Table S3: Strains used in this study

Strain	Genotype, Description	Reference
E. coli DH5α	*E. coli* host for general cloning	General biosystems (Anhui, China)
E. coli BL21 (DE3)	*E. coli* host for protein expression	General biosystems (Anhui, China)
E. coli ET12567/ pUZ8002	Methylation-deficient *E. coli* host for intergeneric conjugation	[9]
Streptomyces clavuligerus CGMCC 4.5336	Streptomyces used to extract the genome as a PCR template	CGMCC
Streptomyces lividans TK64	Host strain for heterologous expression	[10]
Streptomyces coelicolor M1154	Host strain for heterologous expression	[11]
Streptomyces avermitilis SUKA22	Host strain for heterologous expression	[12]
DL10080	*S. lividans* integrated with pSET152 vector	This study
DL10081	*S. lividans* integrated with pLD10101	This study
DL10082	*S. coelicolor* integrated with pSET152 vector	This study
DL10083	*S. coelicolor* integrated with pLD10101	This study
DL10084	*S. avermitilis* integrated with pSET152 vector	This study
DL10085	*S. avermitilis* integrated with pLD10101	This study
DL10086	*S. avermitilis* integrated with pLD10102	This study
DL10087	*S. avermitilis* integrated with pLD10103	This study
DL10088	*S. avermitilis* integrated with pLD10104	This study
DL10089	*S. avermitilis* integrated with pLD10105	This study
DL10090	*S. avermitilis* integrated with pLD10106	This study
DL10091	*S. avermitilis* integrated with pLD10107	This study
DL10092	*E. coli* BL21 harboring pLD10012 and pLD10050	This study
DL10093	*E. coli* ET12567/pUZ8002 harboring pLD10101	This study
DL10094	*E. coli* ET12567/pUZ8002 harboring pLD10102	This study
DL10095	*E. coli* ET12567/pUZ8002 harboring pLD10103	This study
DL10096	*E. coli* ET12567/pUZ8002 harboring pLD10104	This study
DL10097	*E. coli* ET12567/pUZ8002 harboring pLD10105	This study
DL10098	*E. coli* ET12567/pUZ8002 harboring pLD10106	This study
DL10099	*E. coli* ET12567/pUZ8002 harboring pLD10107	This study
DL10100	*E. coli* ET12567/pUZ8002 harboring pLD10108	This study
DL10101	*E. coli* ET12567/pUZ8002 harboring pLD10109	This study

Table S4: Plasmids used in this study.

Plasmid	Description	Reference
pSET152	*E. coli-Streptomyces* integrating vector, including the promoter ermE, Apr^r	[13]
pETDuet-1	Plasmid for cloning, Amp^r	Novagen
pRSFDuet-1	Plasmid for cloning, Kan^r	Novagen
pLD10012	pRSFDuet-1 harboring *ispA, idi, phoN, ipk*	This study
pLD10050	pETDuet-1 harboring *cavC*	This study
pLD10101	pSET152 harboring gene cluster from *S. clavuligerus*	This study
pLD10102	pSET152 harboring *cavB-G*	This study
pLD10103	pSET152 harboring *cavA-G*, except for *cavE*	This study
pLD10104	pSET152 harboring *cavA-F*	This study
pLD10105	pSET152 harboring *cavA-C*	This study
pLD10106	pSET152 harboring *cavBC* and *cavE*	This study
pLD10107	pSET152 harboring *cavBC* and *cavG*	This study

Table S5: Primers used in this study.

Primers	Sequence 5'-3'
S.cla_clu_1F	GGTATCGATAAGCTTGATATCATGTCCGATATATCCTCACAGCCC
S.cla_clu_1R	ATGGTGATCGCTTGCCGATTGCTCGCTG
S.cla_clu_2F	AATCGGCAAGCGATCACCATGATGGCGTG
S.cla_clu_2R	CAGGTCGACTCTAGAGGATCCATGGTGAACGTTCCGGAAGG
sclav_p0068_F	GCGATCGCTGACGTCGGTACCATGTCCCTGAACCACAGCGA
sclav_p0068_R	TTTACCAGACTCGAGGGTACCTCACCGGTGCTCACGCCT
IspA _F	TCATCACCACAGCCAATCCATGGACTTTCCGCAGCAACTC
IspA _R	GATTATGCGGCCGTGTACAATTATTTATTACGCTGGATGATGTAGTCC
IDI_F	TAAGAAGGAGATATACATATGATGCAAACGGAACACGTCATT
IDI_R	GCCGAGCTCGAATTCGGATCCTTATTTAAGCTGGGTAAATGCAGATAA
IUP_F	GCGATCGCTGACGTCGGTACCATGAAGCGCCAGCTGTTTACC
IUP_R	TTTACCAGACTCGAGTTAACGAATAACGGTGCCAATAAA
KnockCavA_1F	GGTATCGATAAGCTTGATATCATGTCCGATATATCCTCACAGCCC
KnockCavA_1R	AAGGCAGGGGGTCCGCACAACCGACCCCGCCGGAC
KnockCavA_2F	TTGTGCGGACCCCCTGCCTTG
KnockCavA_2R	CAGGTCGACTCTAGAGGATCCATGGTGAACGTTCCGGAAGG
PromoterCavBC _F	AGAAGGAGGTTAACACATATGATGGGGATACCTGCGGAACT
PromoterCavBC _R	GAATTTGGTACCGAGCATATGTCACACGAAGCTGAGCCCC
PromoterCavA _F	AGAAGGAGGTTAACAGTGGATTCTGCACGGTCCG
PromoterCavA _R	TTCCTGACTCATAACCTTAAGTCAGGTTGTGGTGGTGATGGT
PromoterCavABC_1F	GGTATCGATAAGCTTGATATCTGTGCGGGCTCTAACACGTC
PromoterCavABC_1R	CCTGCTAGGACCAAAACGAAAAAAGACGC
PromoterCavABC_2F	TTCGTTTTGGTCCTAGCAGGGCTCCAAAACTAACG
PromoterCavABC_2R	CAGGTCGACTCTAGAGGATCCAAAGCAAGCAAAGAAAAAAGGC

Table S6: Summary of ^{1}H NMR (400 MHz) and ^{13}C NMR (100 MHz) data of compound **8** in $CDCl_3$ (δ in ppm, J in Hz).

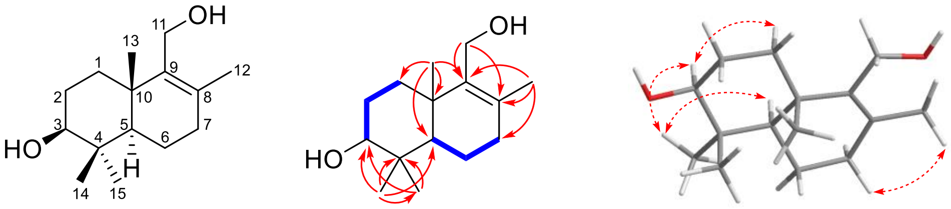

Chemical structure of **8** and ^{1}H-^{1}H COSY (▬), key HMBC correlations (→), and key ROESY correlations (⇠⇢).

No.	δ_C, type	δ_H, mult. (J, Hz)
1a	35.0, CH_2	1.43, m
1b		1.92, dt (13, 3.5)
2a	27.8, CH_2	1.67, m
2b		1.75, m
3	79.0, C	3.26, dd (11.6, 4.7)
4	38.9, C	
5	51.0, CH	1.13, dd (12.5, 2.0)
6a	18.74, CH_2	1.49, m
6b		1.68, m
7a	71.1, CH_2	2.07, d (4.2)
7b		2.10, d (5.9)
8	132.9, C	
9	140.4, C	
10	38.0, C	
11a	58.4, CH_2	4.04, d (11.5)
11b		4.21, d (11.5)
12	19.3, CH_3	1.72, s
13	20.8, CH_3	0.97, s
14	28.2, CH_3	1.01, s
15	15.6, CH_3	0.81, s

Table S7: Summary of ^{1}H NMR (400 MHz) and ^{13}C NMR (100 MHz) data of compound **9** in DMSO-*d4* (δ in ppm, J in Hz).

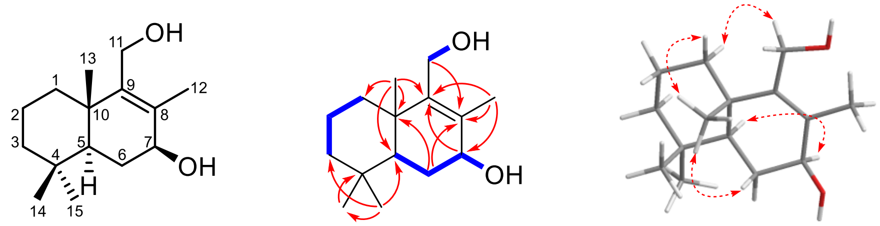

Chemical structure of **9** and ^{1}H-^{1}H COSY (blue line), key HMBC correlations (red arrow), and key ROESY correlations (red dashed double arrow).

No.	δ_C, type	δ_H, mult. (J, Hz)
1a	35.9, CH_2	1.22, m
1b		1.77, d (13)
2a	18.1, CH_2	1.41, dt (15.3, 2.8)
2b		1.56, dt (13.4, 3.4)
3a	41.2, CH_2	1.12, m
3b		1.35, d (2.6)
4	32.5, C	
5	49.5, CH	1.07, m
6a	29.4, CH_2	1.31, m
6b		1.84, m
7	71.1, CH	3.79, t (5.7)
8	133.4, C	
9	142.0, C	
10	38.3, C	
11a	56.3, CH_2	3.83, d (5.4)
11b		3.92, dd (11.4, 4.5)
12	14.9, CH_3	1.65, d (0.9)
13	20.5, CH_3	0.96, s
14	33.0, CH_3	0.85, s
15	21.5, CH_3	0.81, s

Figure S1: ^{1}H NMR spectrum of 3*β*-hydroxy-drimenol (**2**) in CD_3OD (400 MHz).

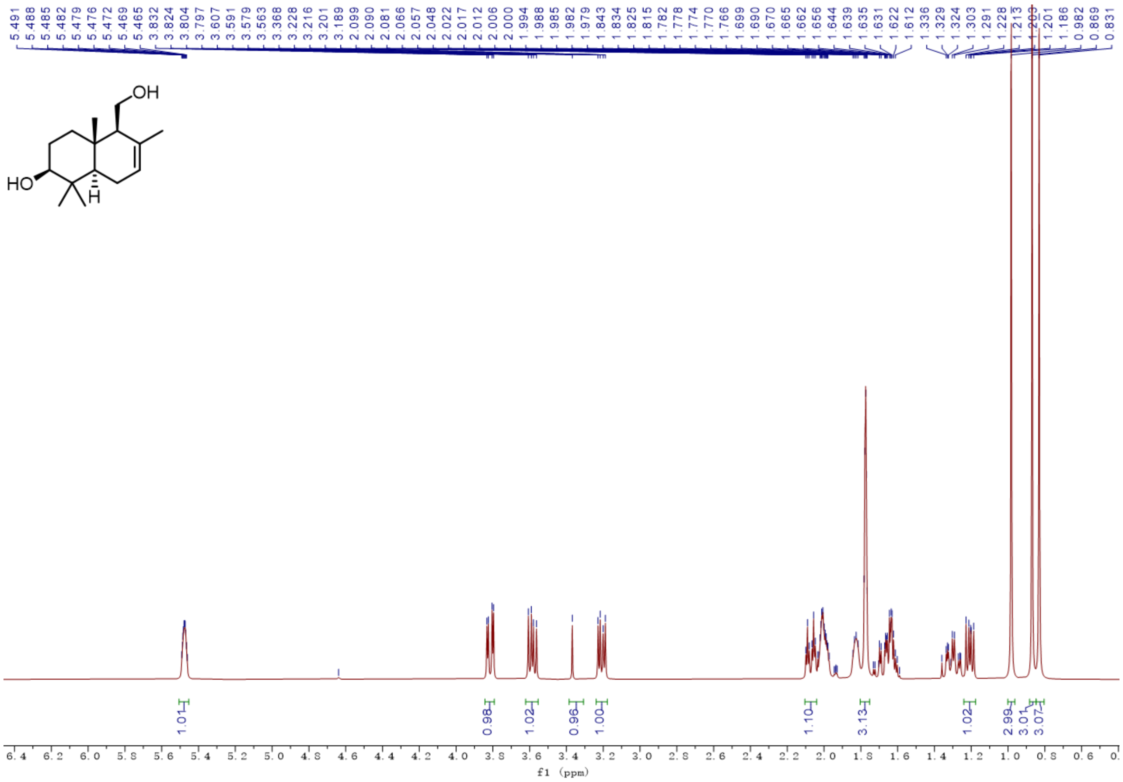

Figure S2: ^{13}C NMR spectrum of 3*β*-hydroxy-drimenol (**2**) in CD_3OD (100 MHz).

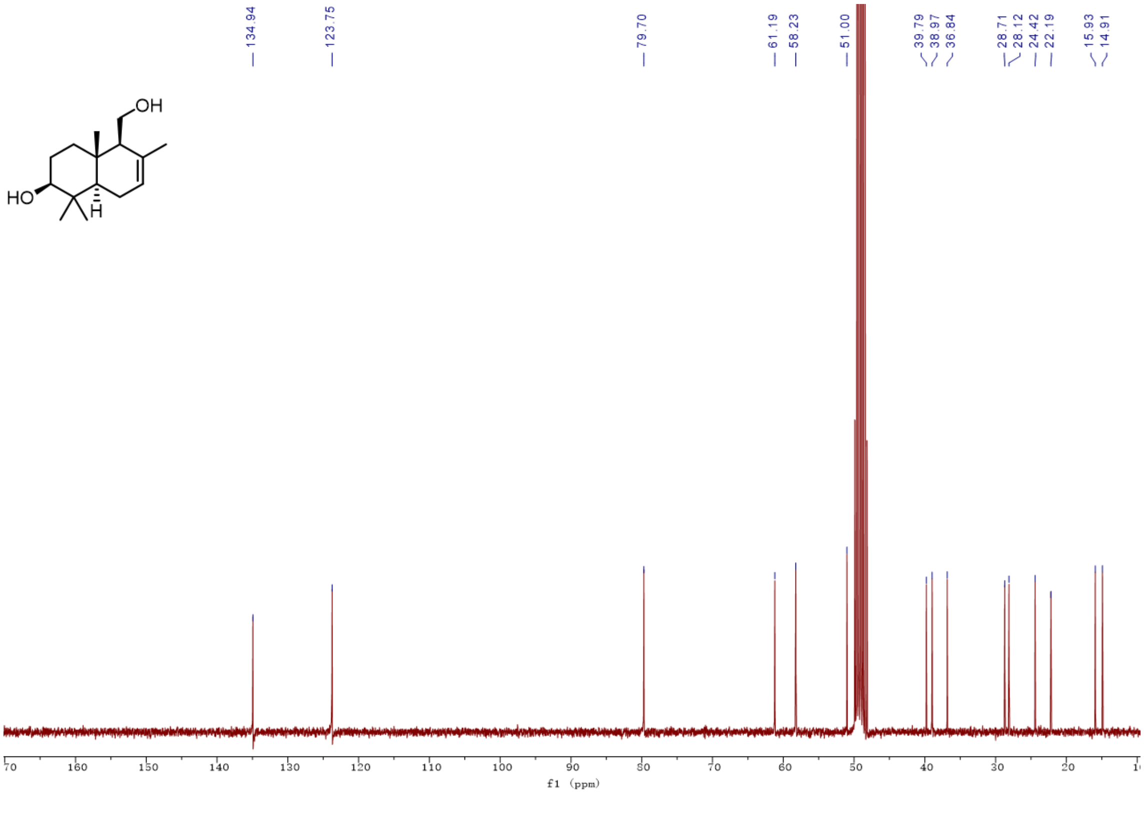

Figure S3: DEPT 135° spectrum of 3β-hydroxy-drimenol (**2**) in CD_3OD.

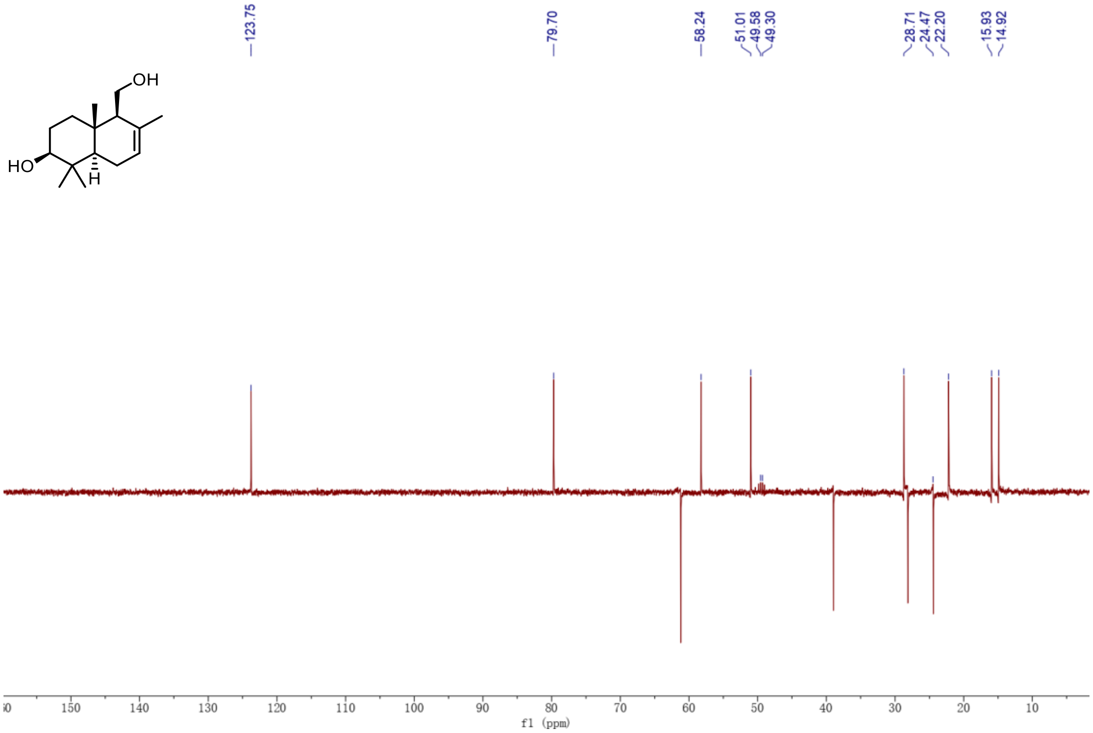

Figure S4: ^{1}H NMR spectrum of 2α-hydroxy-drimenol (**3**) in $CDCl_3$ (400 MHz).

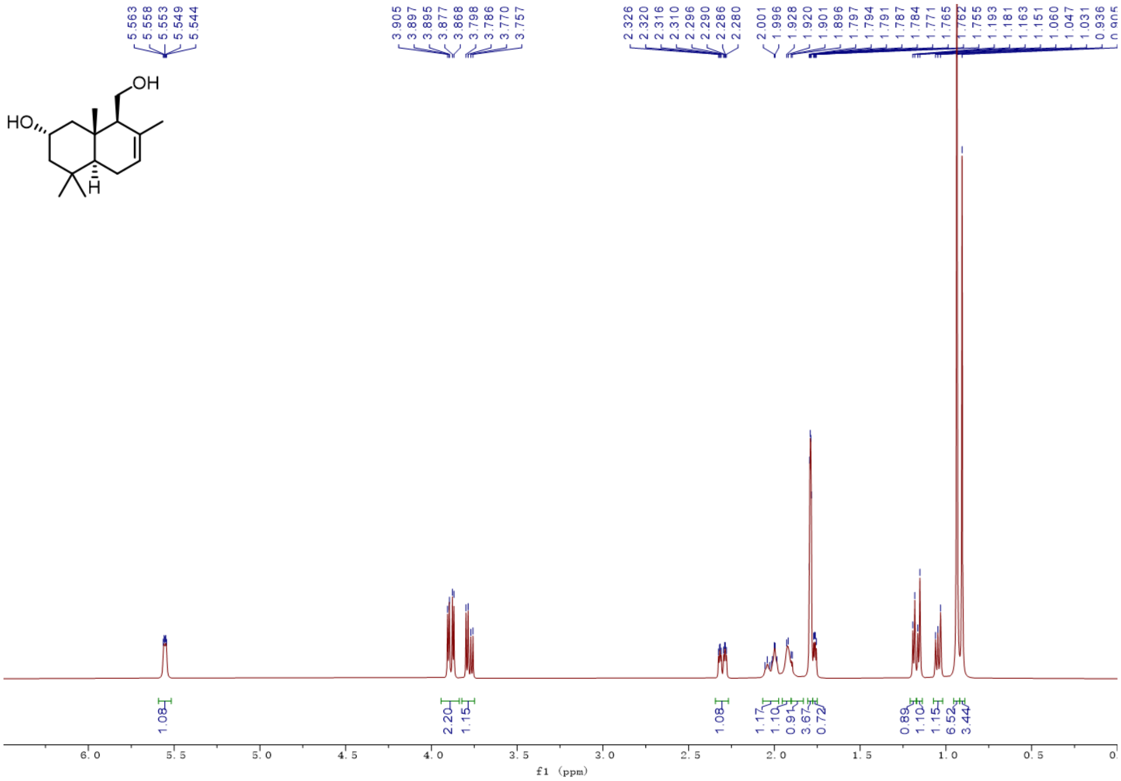

Figure S5: ^{13}C NMR spectrum of 2*α*-hydroxy-drimenol (**3**) in $CDCl_3$ (100 MHz).

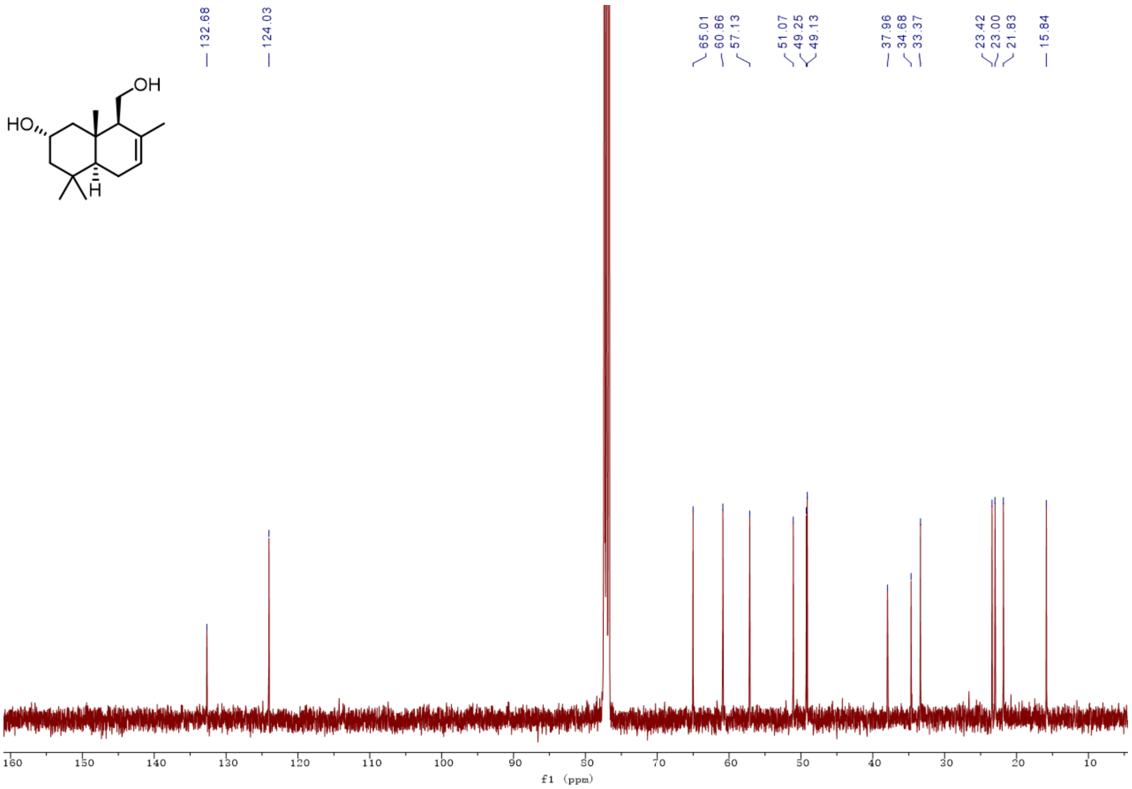

Figure S6: ^{1}H NMR spectrum of 3-keto-drimenol (**4**) in CD_3OD (400 MHz).

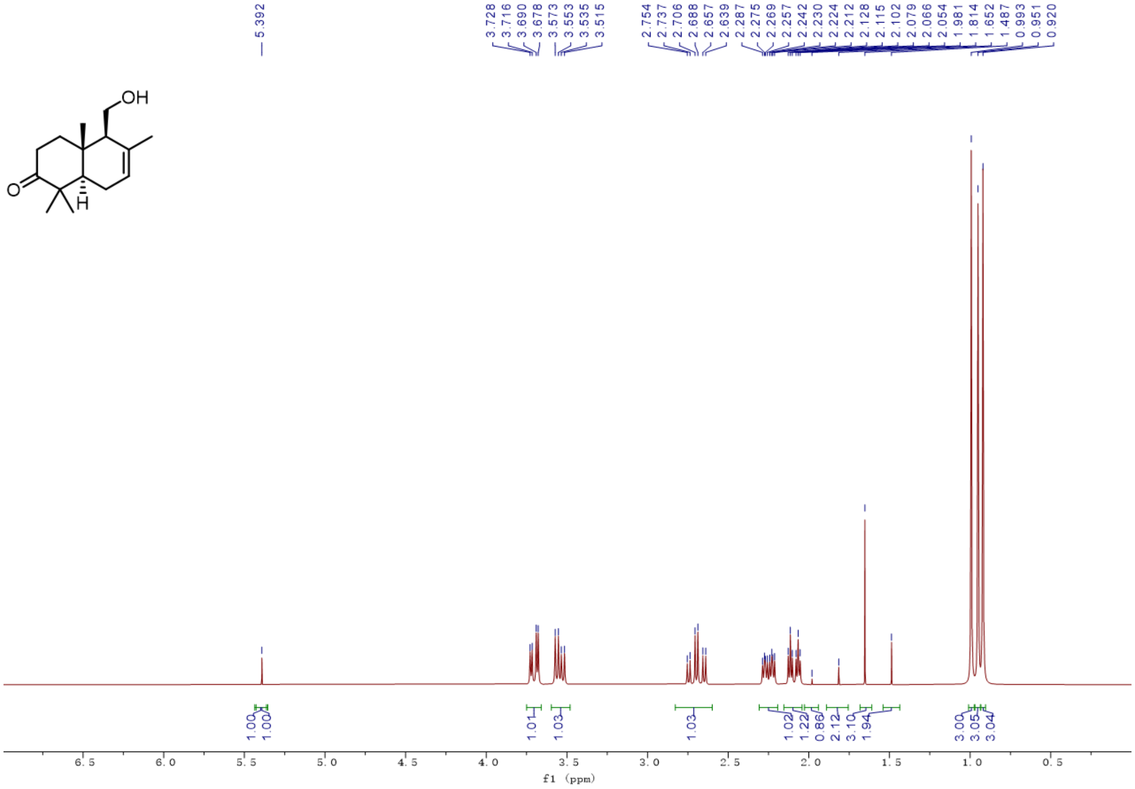

Figure S7: ^{13}C NMR spectrum of 3-keto-drimenol (**4**) in CD_3OD (100 MHz).

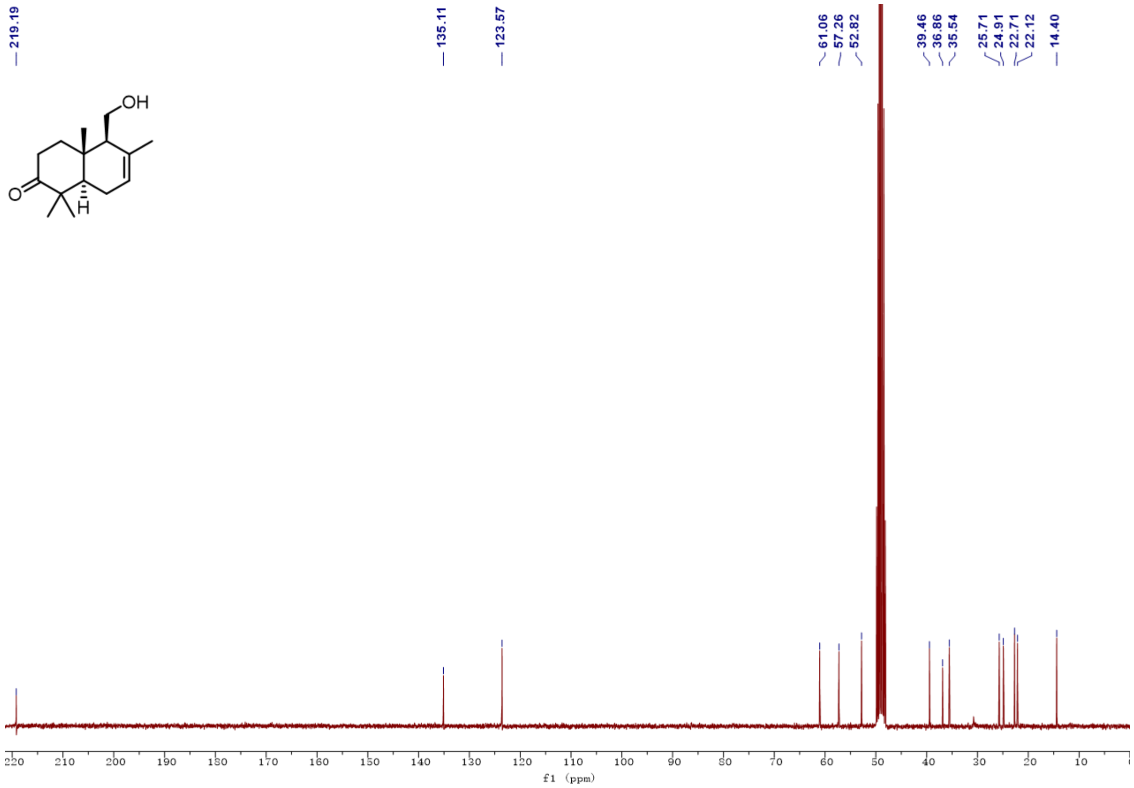

Figure S8: Sequence alignment of SsDMS and CavC.

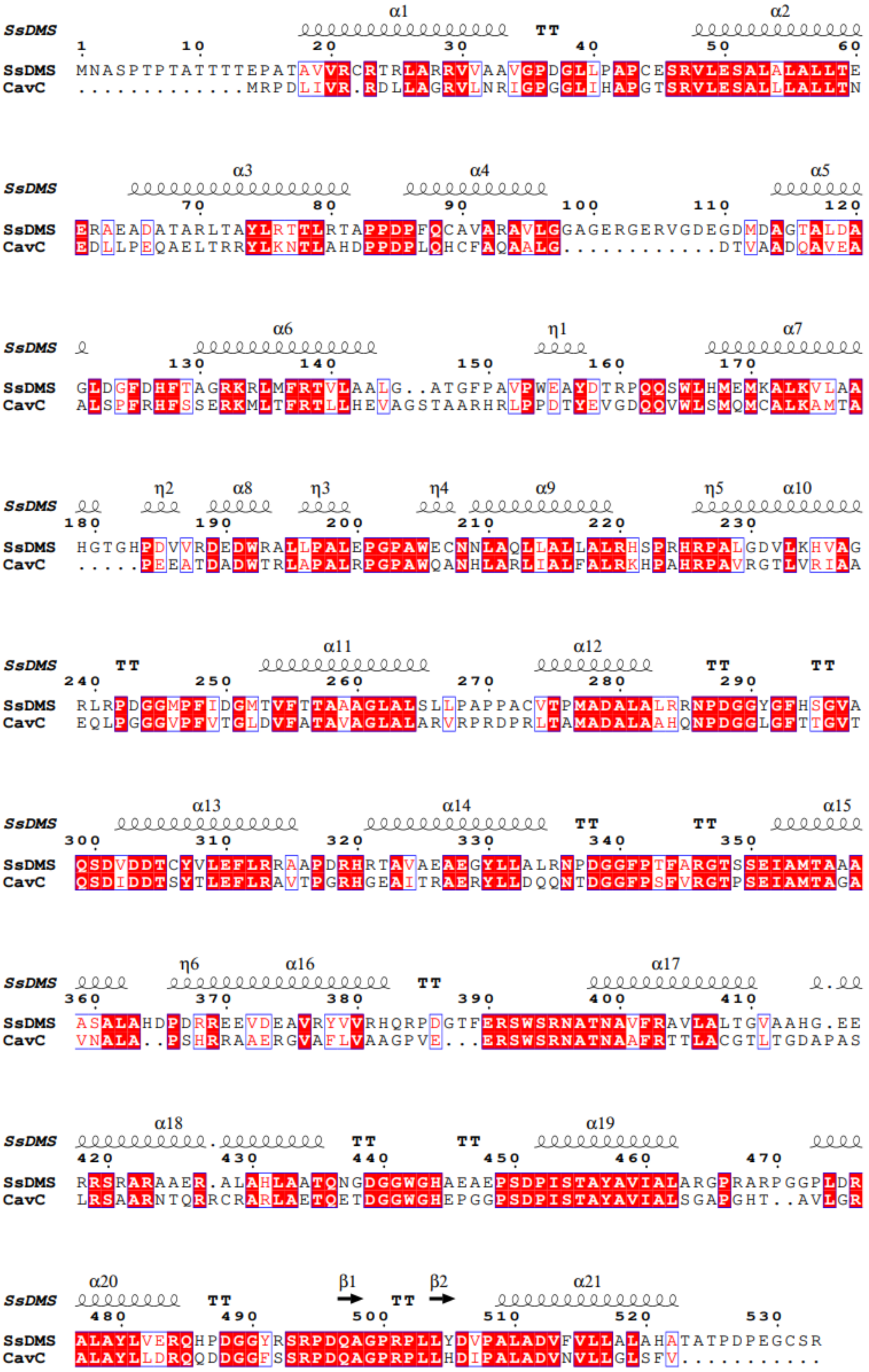

Figure S9: *In vivo* characterization of drimenyl diphosphate synthase of CavC. The HPLC profile showed that *E. coli* DL10092 strain, harboring the *cavC* gene and the truncated artificial FPP-overproduction system, efficiently produced a substantial quantity of drimenol in comparison to the wild-type control of *E. coli* BL21 (DE3).

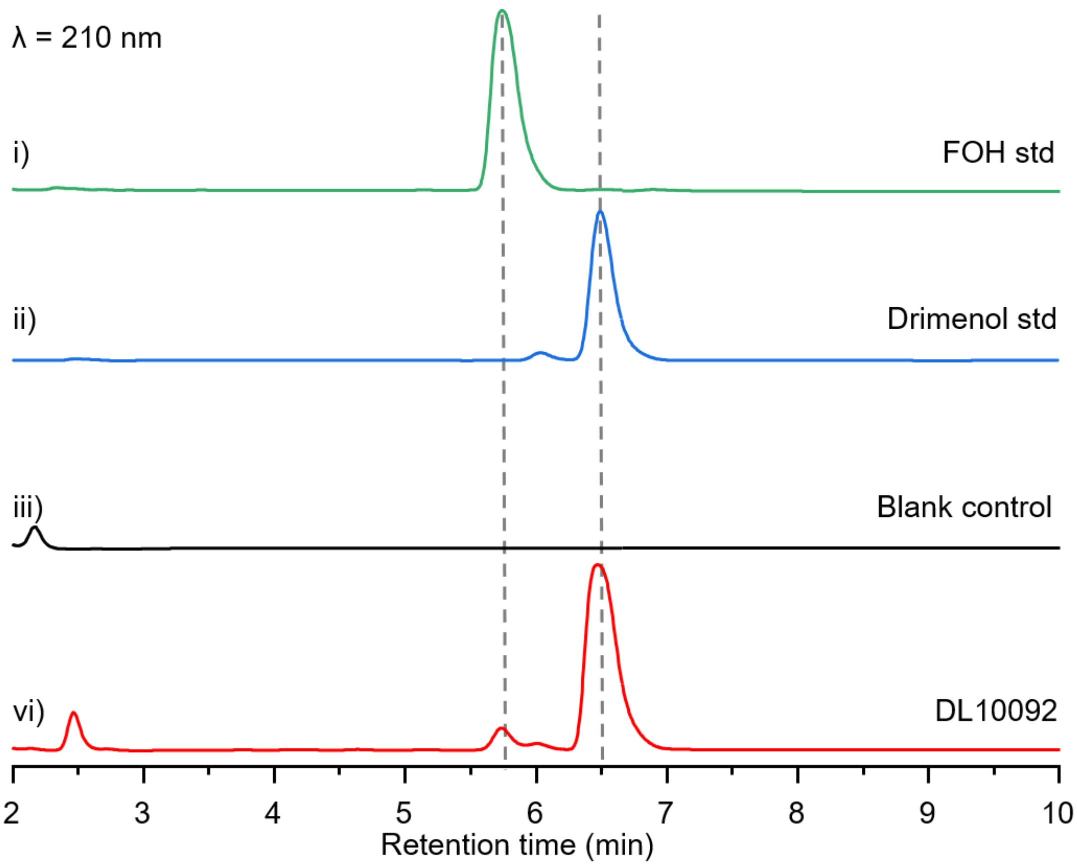

Figure S10: HPLC analysis of metabolites of engineered *S. lividans* TK64 DL10081 in PTMM、XTM, and ISM3 media. *S. lividans* TK64 with empty pSET152 was used as a control.

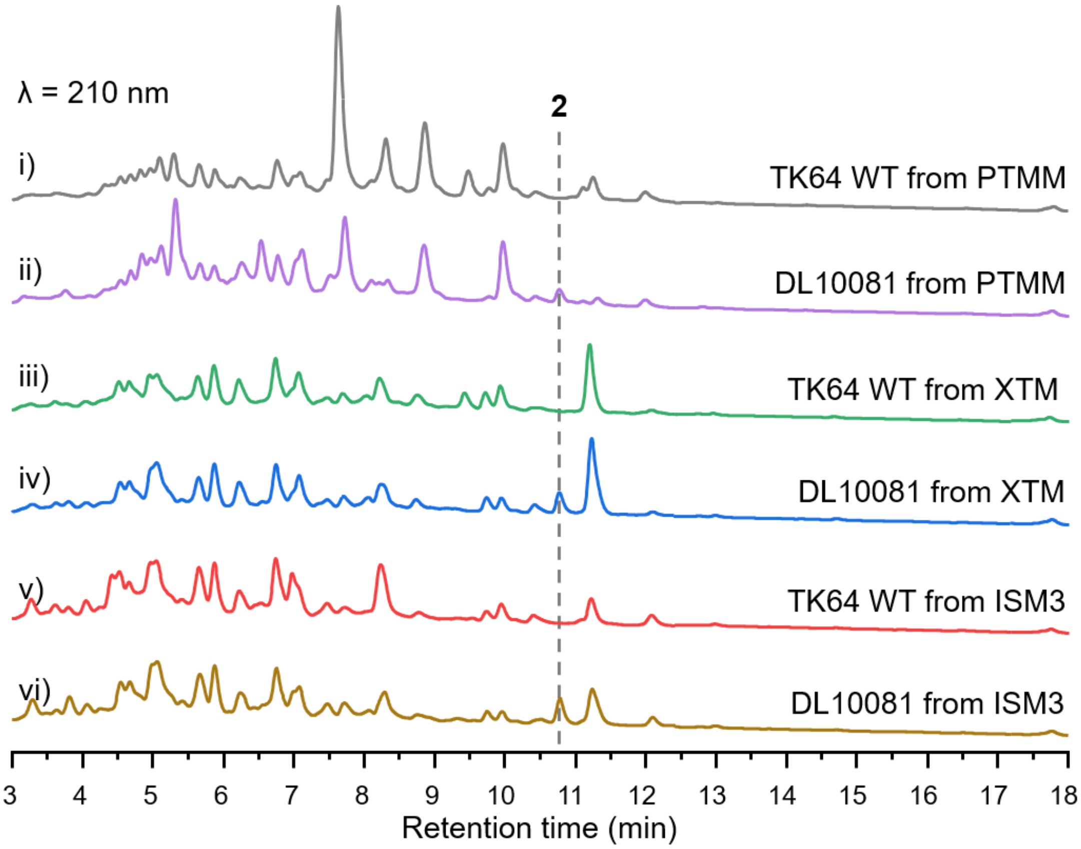

Figure S11: ^{1}H NMR spectrum of 3β-hydroxy-albicanol (**7**) in $CDCl_3$ (400 MHz).

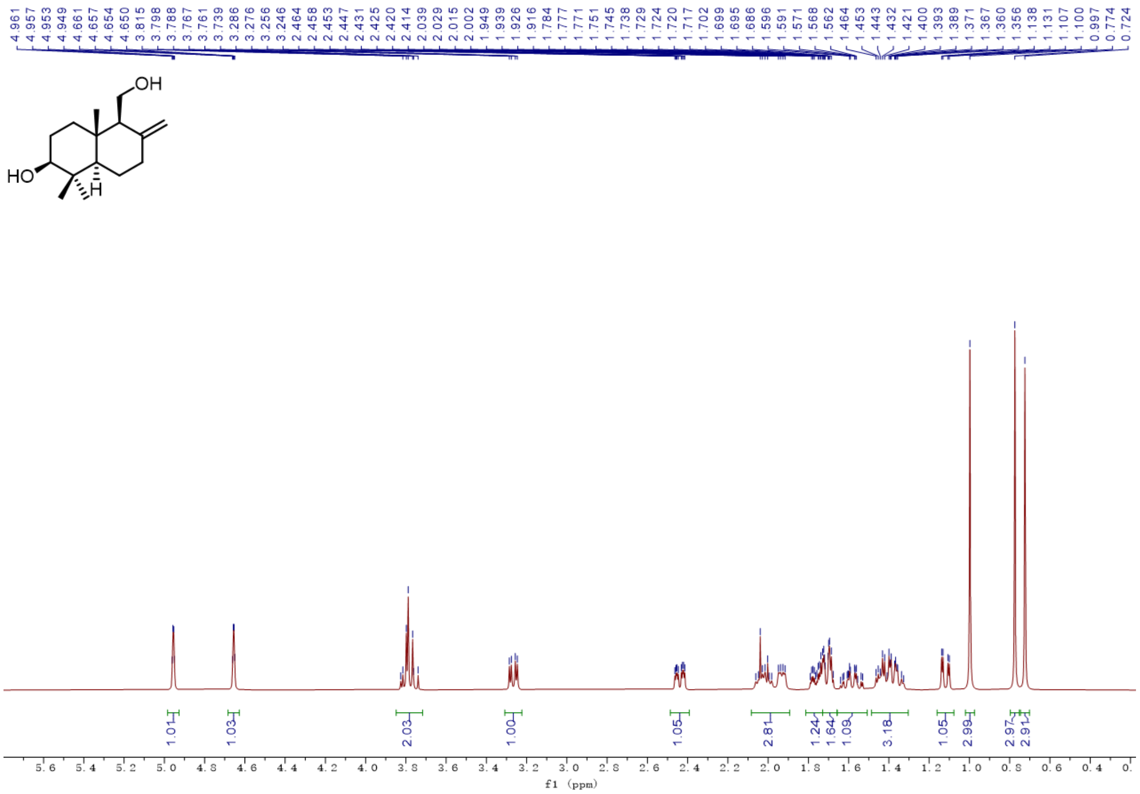

Figure S12: ^{13}C NMR spectrum of 3β-hydroxy-albicanol (**7**) in $CDCl_3$ (100 MHz).

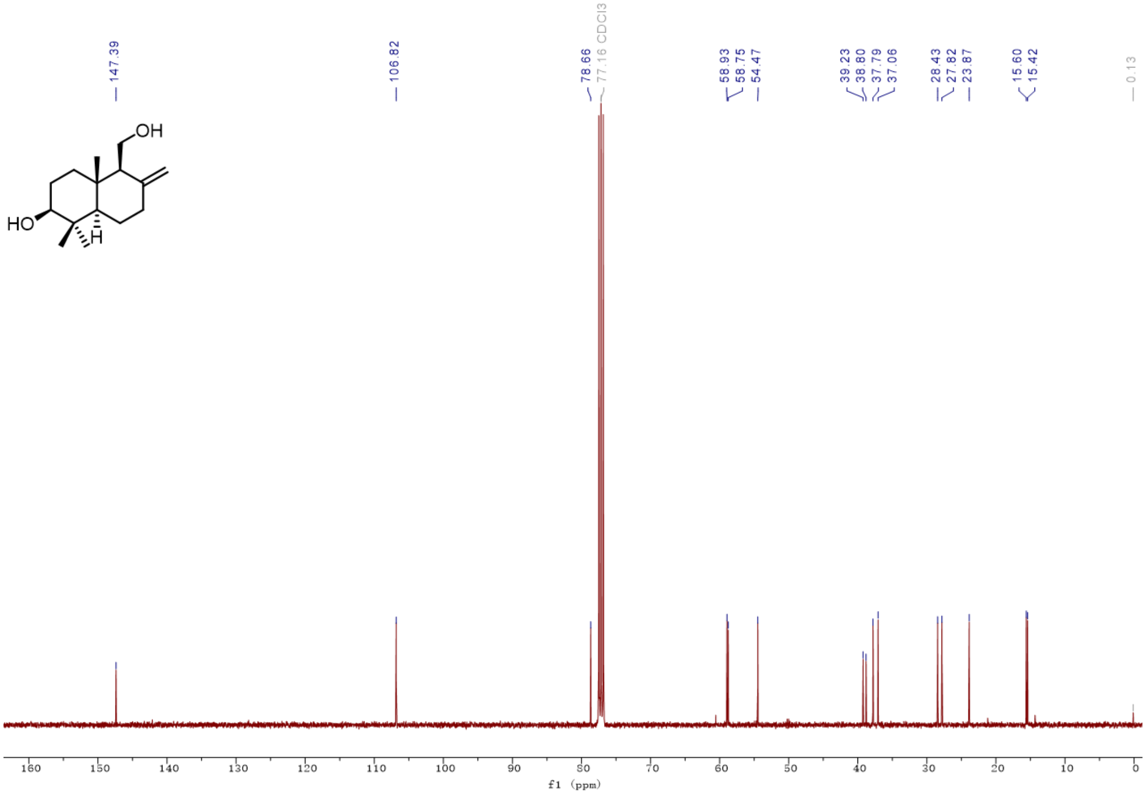

Figure S13: ^{1}H NMR spectrum of 3β-hydroxy-drim-8-ene-11-ol (**8**) in $CDCl_3$ (400 MHz).

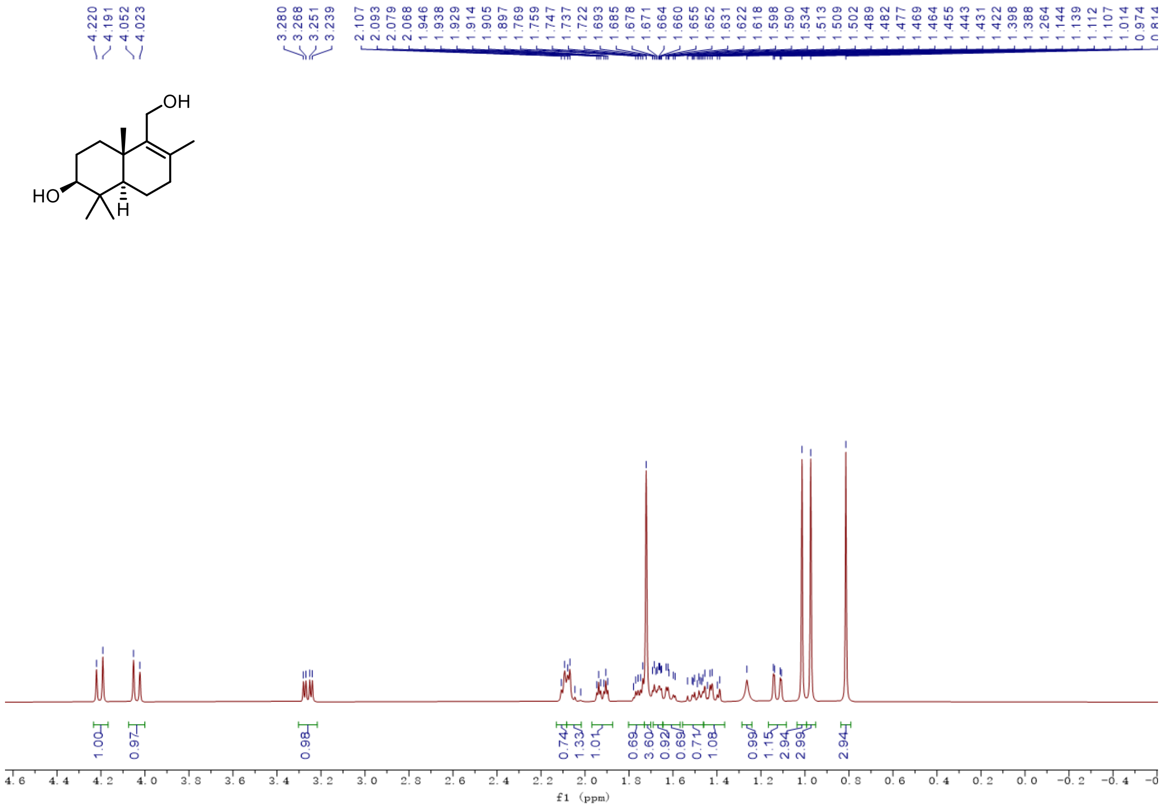

Figure S14: ^{13}C NMR spectrum of 3β-hydroxy-drim-8-ene-11-ol (**8**) in $CDCl_3$ (100 MHz).

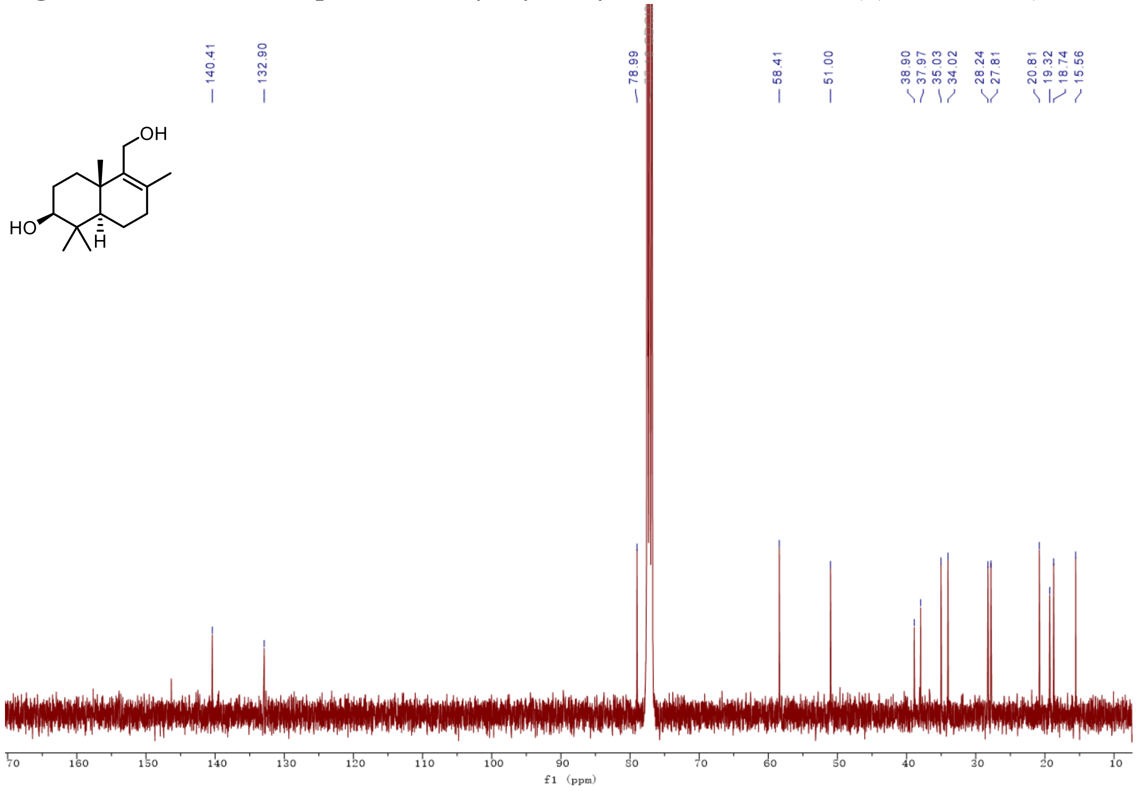

Figure S15: HMBC spectrum of 3*β*-hydroxy-drim-8-ene-11-ol (**8**) in $CDCl_3$.

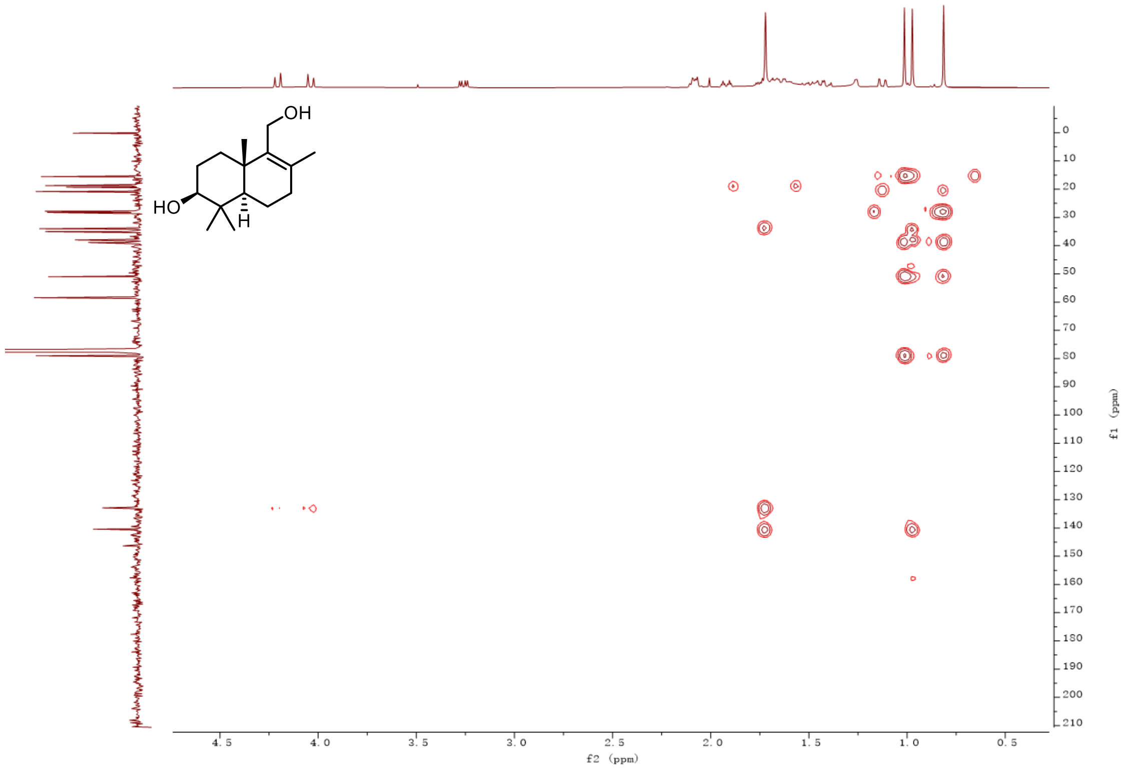

Figure S16: HSQC spectrum of 3*β*-hydroxy-drim-8-ene-11-ol (**8**) in $CDCl_3$.

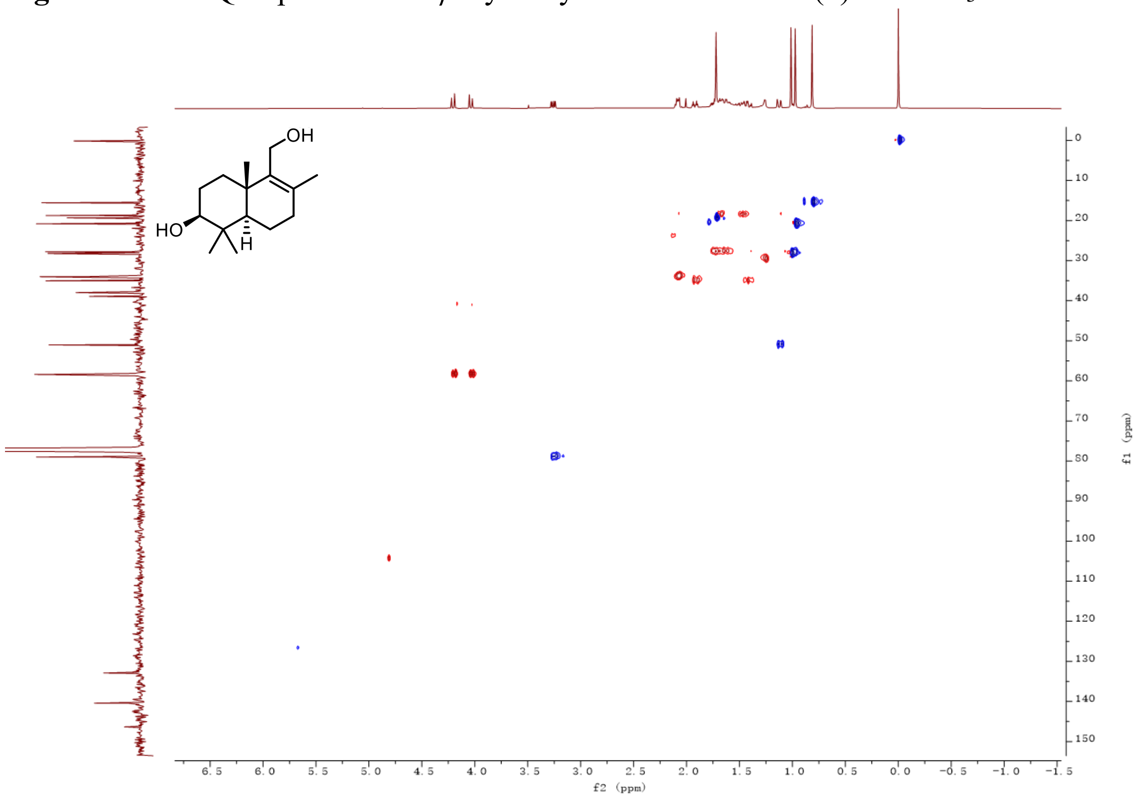

Figure S17: ^{1}H-^{1}H COSY spectrum of 3β-hydroxy-drim-8-ene-11-ol (**8**) in $CDCl_3$.

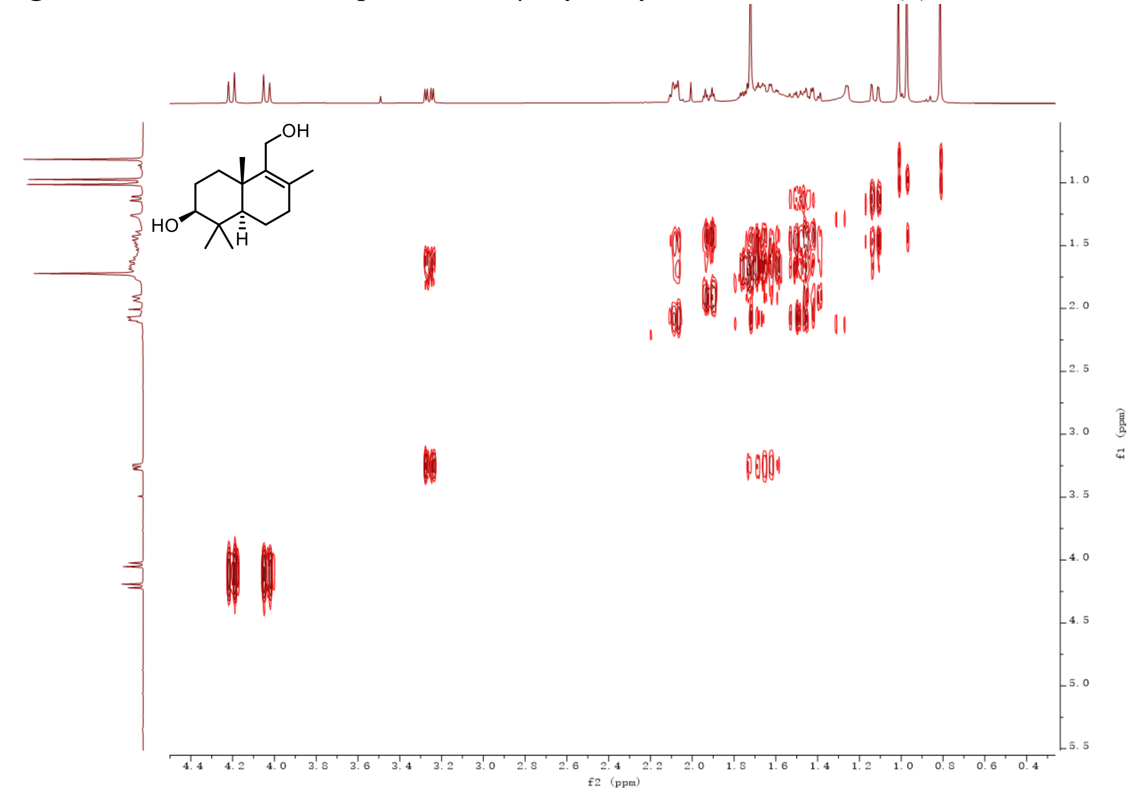

Figure S18: ROESR spectrum of 3β-hydroxy-drim-8-ene-11-ol (**8**) in $CDCl_3$.

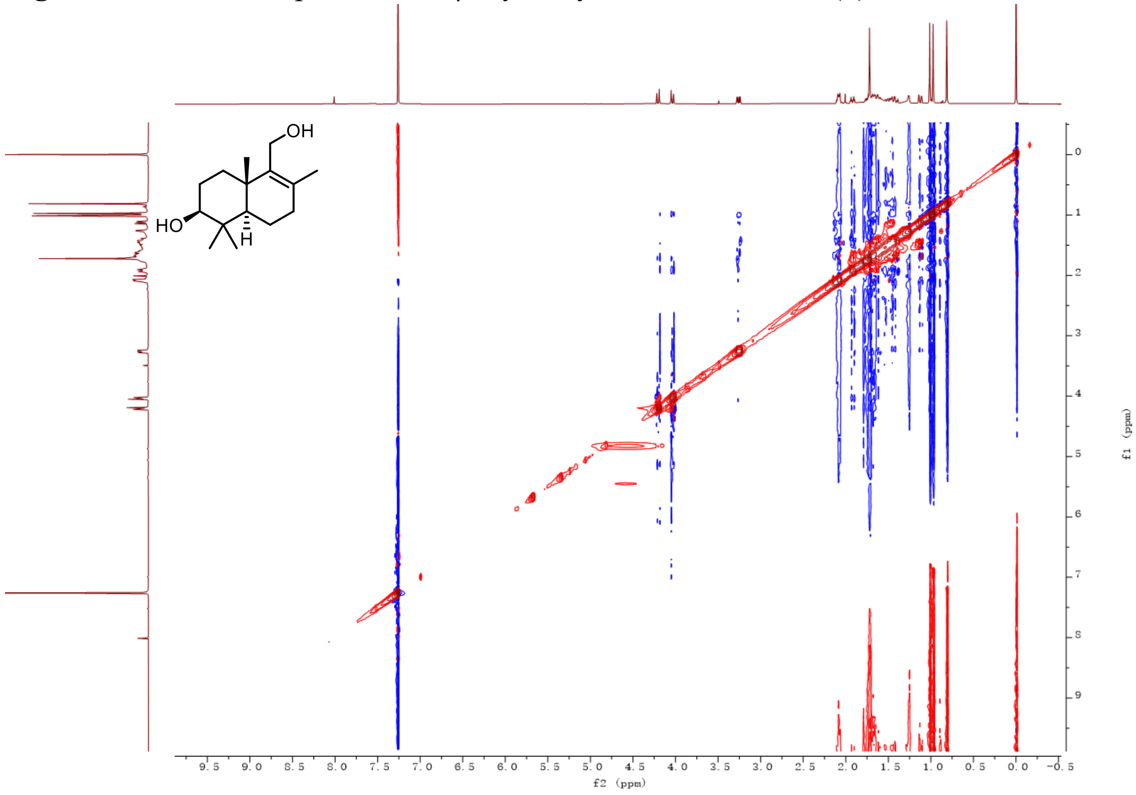

Figure S19. HRESIMS (negative mode) spectrum of 7β-hydroxy-drim-8-ene-11-ol (**9**).

Qualitative Analysis Report

**Data Filename**	8.11-1-.d	**Sample Name**	8.11-1
**Sample Type**	Sample	**Position**	P1-D1
**Instrument Name**	Instrument 1	**User Name**	
**Acq Method**	E-.m	**Acquired Time**	12/21/2023 15:13:26 (UTC+08:00)
**IRM Calibration Status**	Success	**DA Method**	QualDAMethod.m
**Comment**			

**Sample Group**		**Info.**	
**Stream Name**	LC 1	**Acquisition Time (Local)**	12/21/2023 15:13:26 (UTC+08:00)
**Acquisition SW Version**	6200 series TOF/6500 series Q-TOF 10.1 (48.0)	**QTOF Driver Version**	10.01.00
**QTOF Firmware Version**	27.811	**Tune Mass Range Max.**	3200

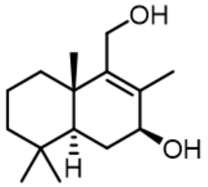

Spectra

Fragmentor Voltage	Collision Energy	Ionization Mode
175	0	ESI

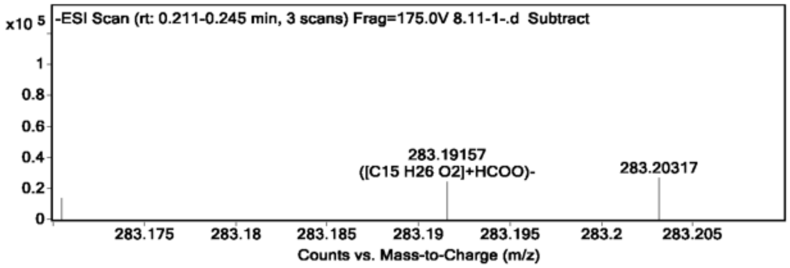

Peak List

m/z	z	Abund
332.28137	1	2387602.75
339.23421	1	5273981.5
600.3905	1	1377520.75
713.47474	1	1764773.63
723.50369	1	1955254.13

Formula Calculator Element Limits

Element	Min	Max
C	14	16
H	24	28
O	1	3

Formula Calculator Results

Formula	Best	Mass	Tgt Mass	Diff (ppm)	Ion Species	CalculatedMz
C15 H26 O2	TRUE	238.19289	238.19328	1.64	C16 H27 O4	283.19148

--- End Of Report ---

Figure S20:^{1}H NMR spectrum of 7*β*-hydroxy-drim-8-ene-11-ol (**9**) in DMSO-d_6 (400 MHz).

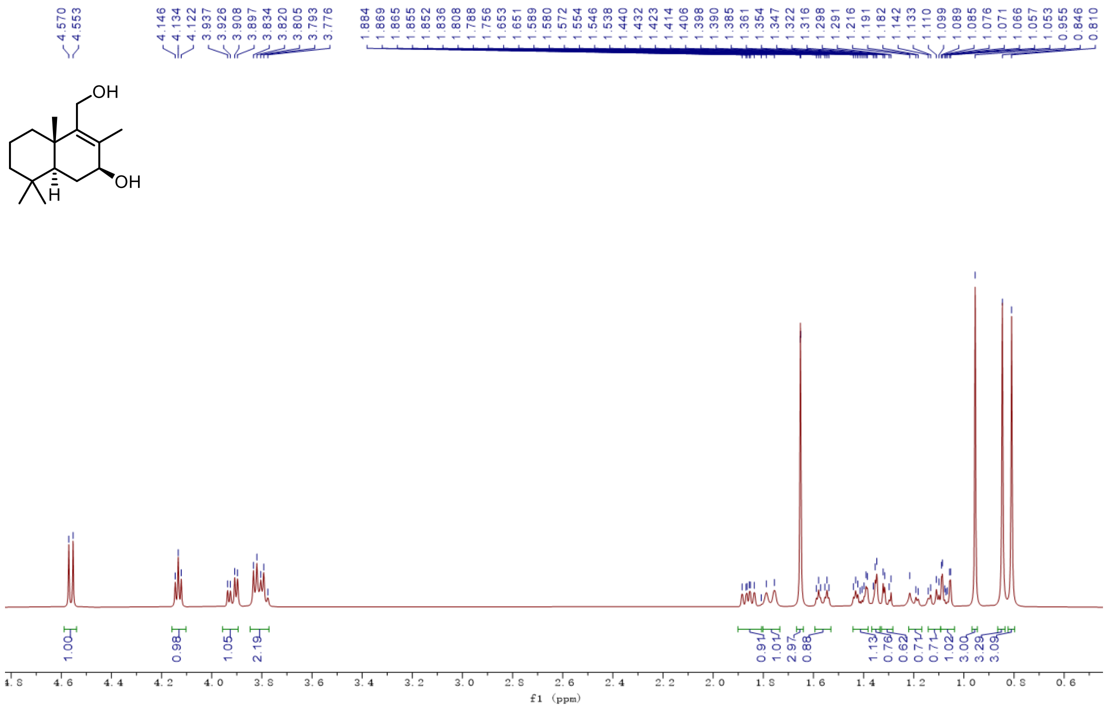

Figure S21:^{13}C NMR spectrum of 7*β*-hydroxy-drim-8-ene-11-ol (**9**) in DMSO-d_6 (100 MHz).

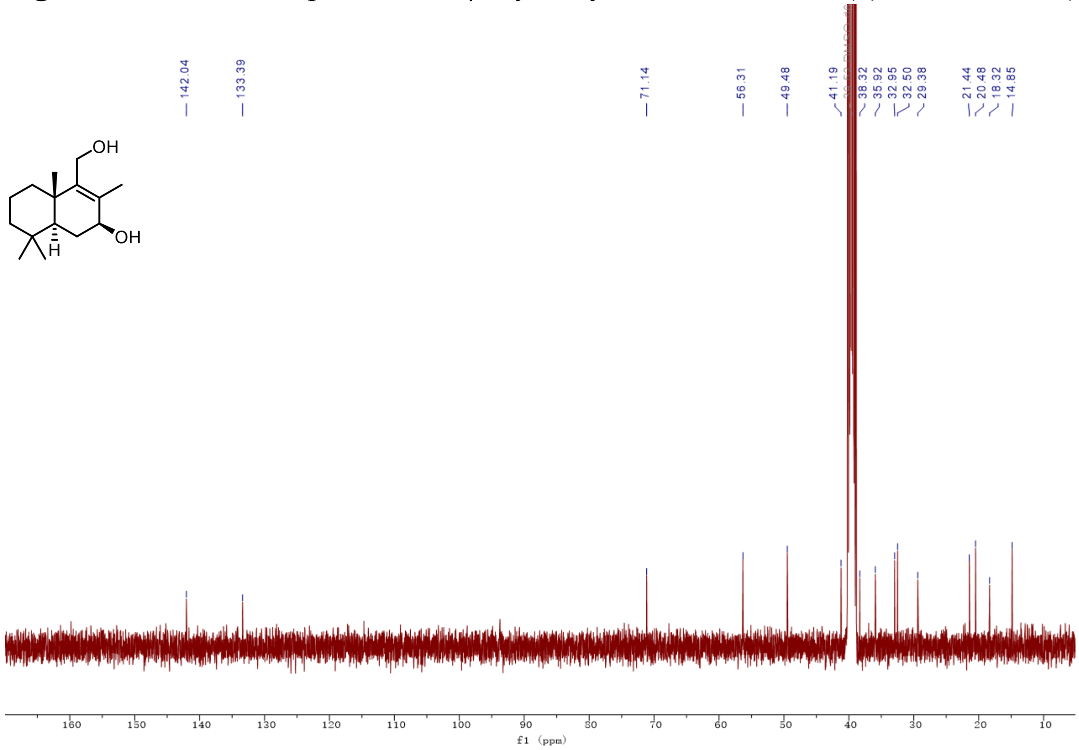

Figure S22: DEPT 135° spectrum of 7β-hydroxy-drim-8-ene-11-ol (**9**) in DMSO-d_6.

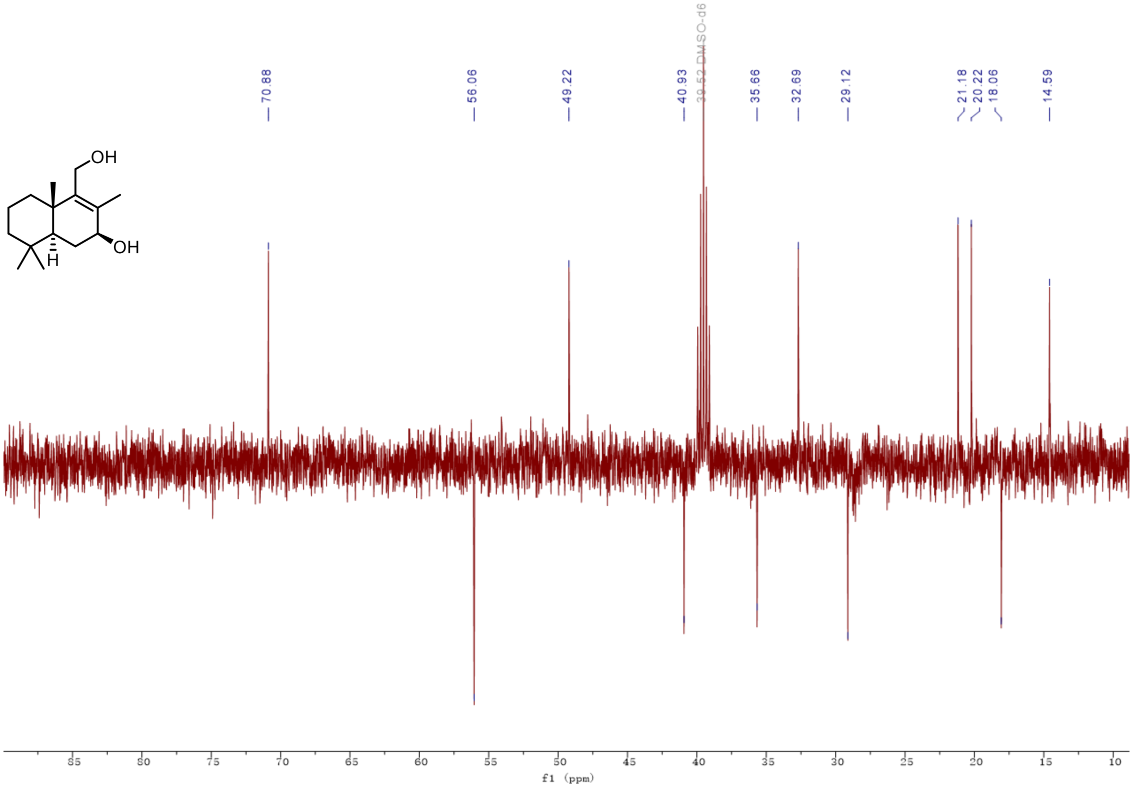

Figure S23: HMBC spectrum of 7β-hydroxy-drim-8-ene-11-ol (**9**) in DMSO-d_6.

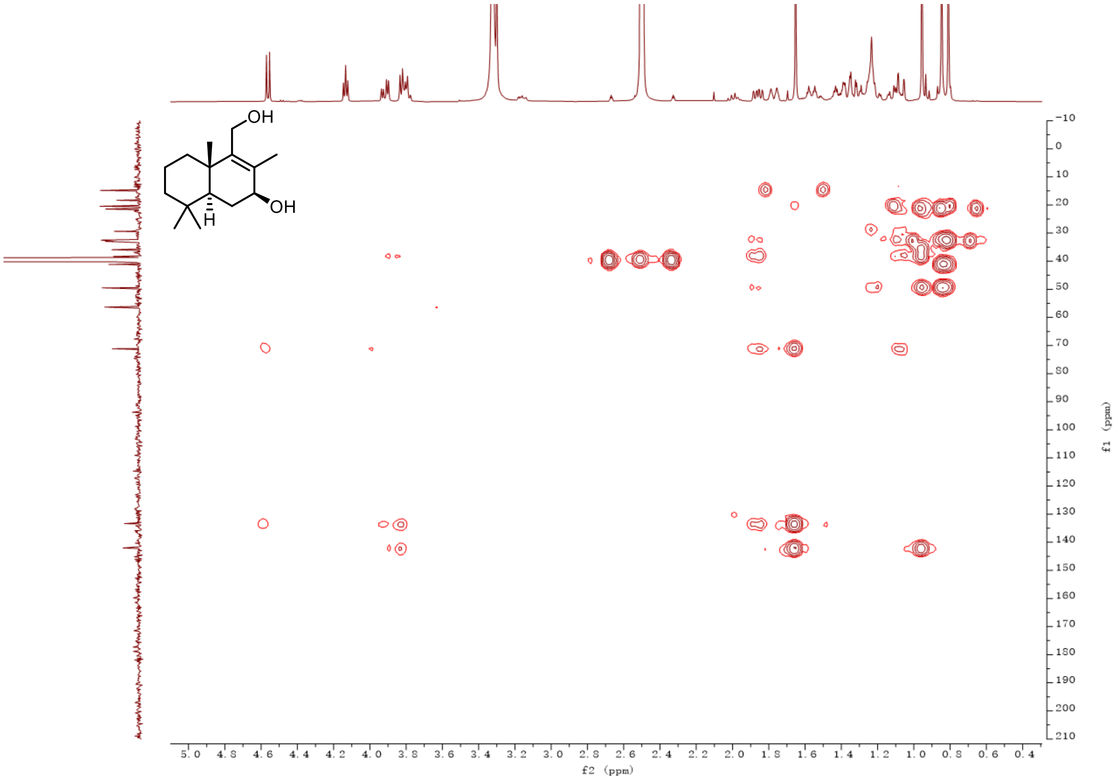

Figure S24: HSQC spectrum of 7β-hydroxy-drim-8-ene-11-ol (**9**) in DMSO-d_6.

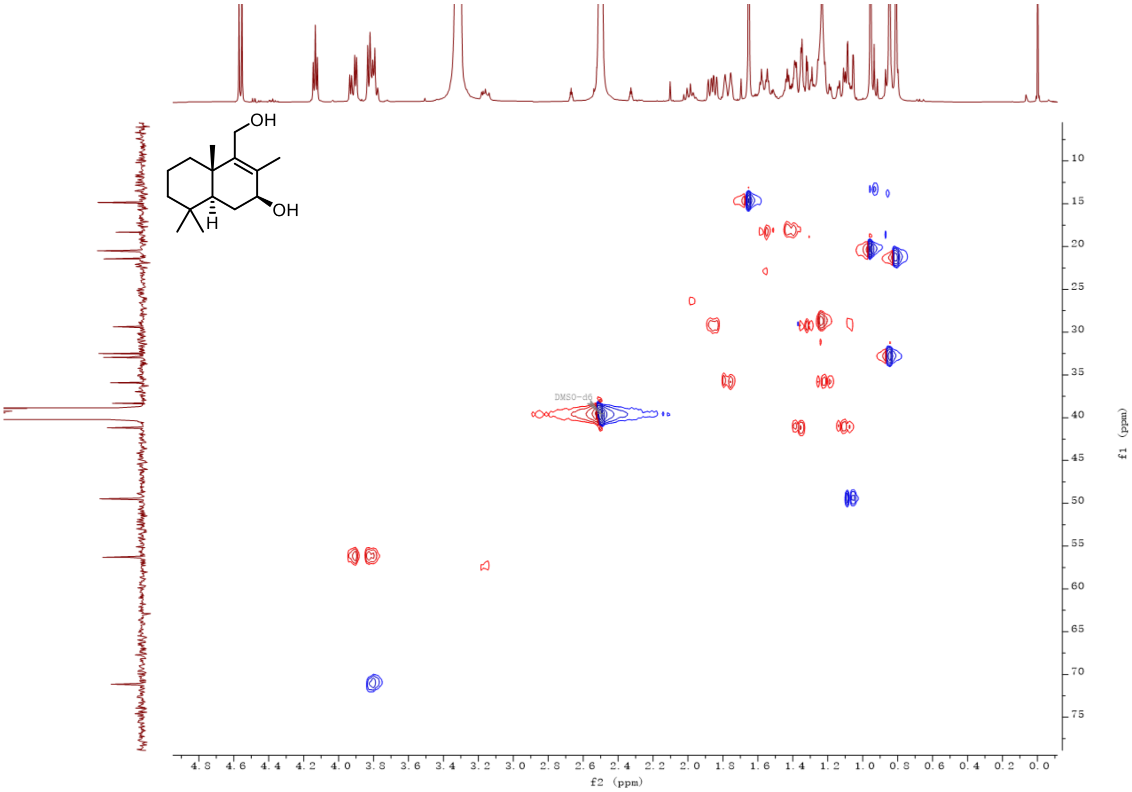

Figure S25: ^{1}H-^{1}H COSY spectrum of 7β-hydroxy-drim-8-ene-11-ol (**9**) in DMSO-d_6.

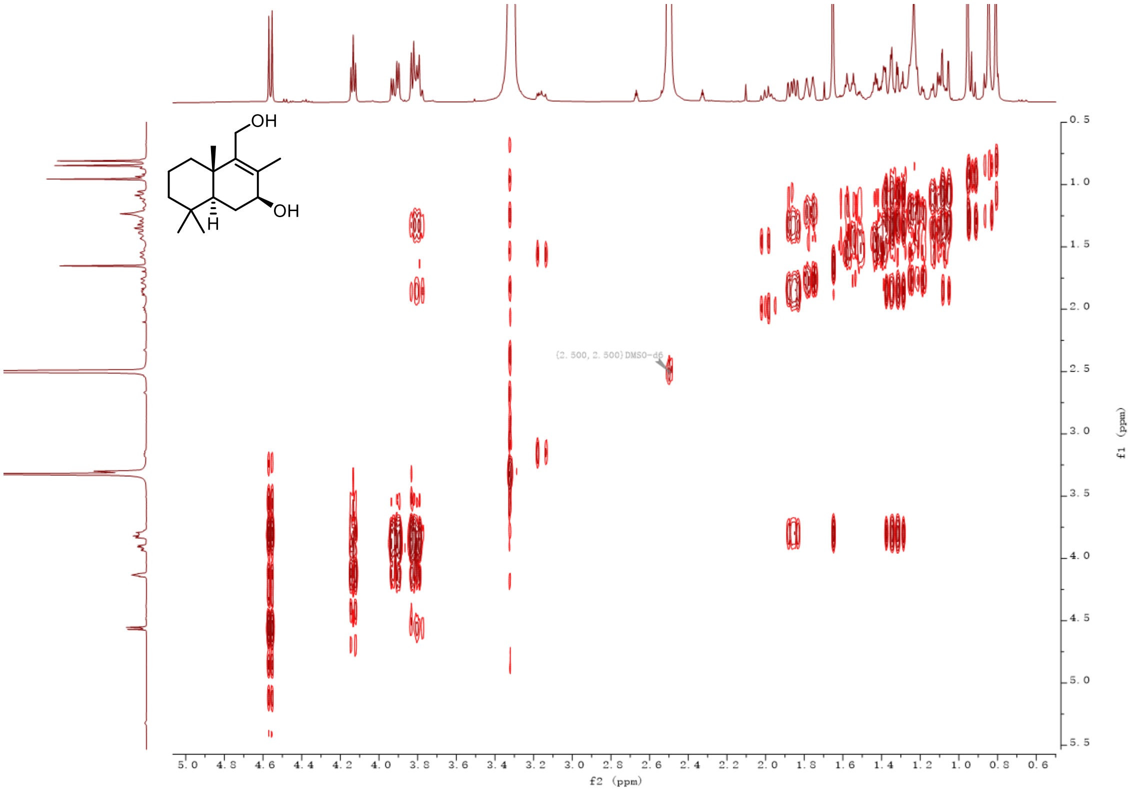

Figure S26: ROESY spectrum of 7β-hydroxy-drim-8-ene-11-ol (**9**) in DMSO-d_6.

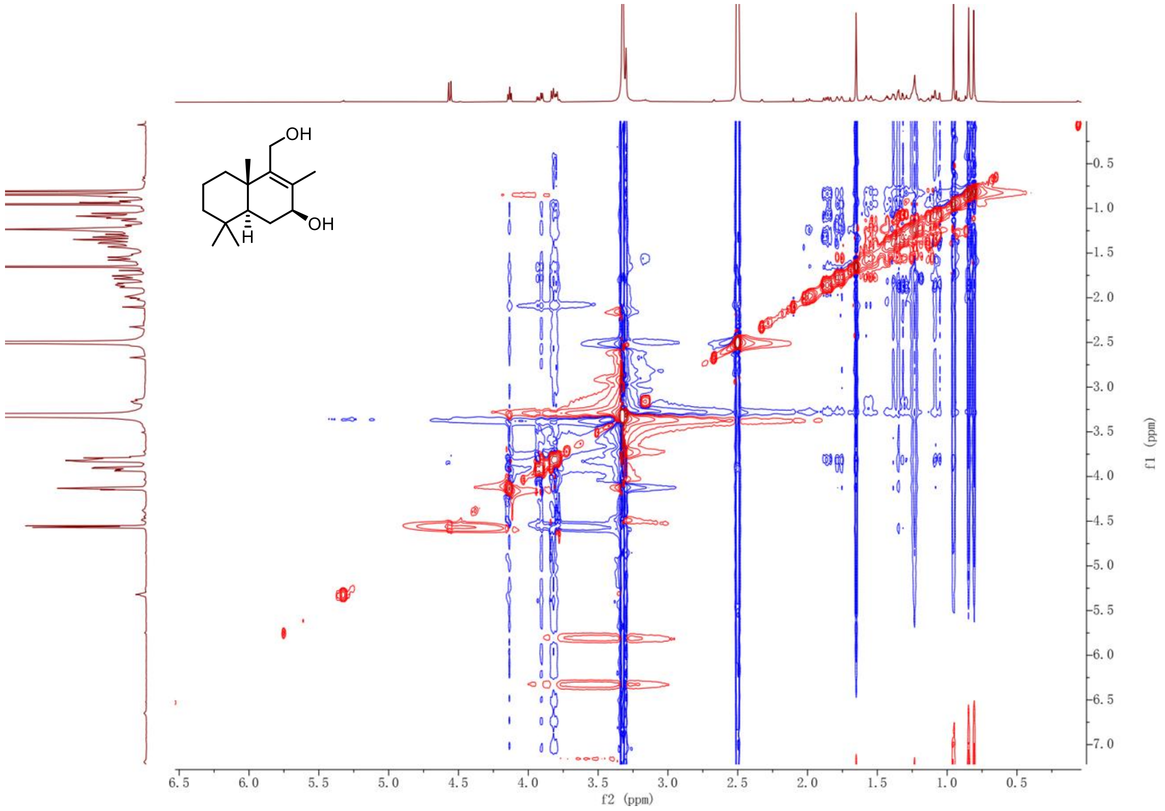

References

1. Zallot, R.; Oberg, N.; Gerlt, J. A. *Biochemistry. U.S.* **2019**, *58*, 4169–4182. doi: 10.1021/acs.biochem.9b00735
2. Blin, K.; Shaw, S.; Kloosterman, A. M.; Charlop-Powers, Z.; van Wezel, G. P.; Medema, M. H.; Weber, T. *Nucleic. Acids. Res.* **2021**, *49*, W29–W35. doi: 10.1093/nar/gkab335
3. Dong, L.-B.; Zhang, X.; Rudolf, J. D.; Deng, M.-R.; Kalkreuter, E.; Cepeda, A. J.; Renata, H.; Shen, B.; *J. Am. Chem. Soc.* **2019**, *141*, 4043–4050. doi: 10.1021/jacs.8b13452
4. Dong, L.-B.; Rudolf, J. D.; Kang, D.; Wang, N.; He, C. Q.; Deng, Y.; Huang, Y.; Houk, K. N.; Duan, Y.; Shen, B. *Nat. Commun*. **2018**, *9*, 2362. doi: 10.1038/s41467-018-04747-y
5. Hsu, S. Y.; Perusse, D.; Hougard, T.; Smanski, M. J. *ACS Synth. Biol*. **2019**, *8*, 2397– 2403, doi:10.1021/acssynbio.9b00261
6. Pines, G.; Freed, E. F.; Winkler, J. D.; Gill, R. T. *ACS Synth. Biol*. **2015**, *4*, 1176-1185. doi:10.1021/acssynbio.5b00009
7. Xu, D.; Sheng, Y.; Zhou, Z.-Y.; Liu, R.; Leng, Y.; Liu, J. K. *Chem. Pharm. Bull*. **2009**, *57*, 433-435. doi: 10.1021/acssynbio.5b00009
8. Pu, X.-J.; Hu, Q.-Y.; Li, S.-S.; Li, G.-H.; Zhao, P.-J. *Phytochemistry*. **2021**, *189*, 112852. doi: 10.1016/j.phytochem
9. Otaka, J.; Araya, H. *Phytochem Lett*. **2013**, *6*, 598–601. doi: 10.1016/j.phytol.2013.07.010
10. Paget, M. S.; Chamberlin, L.; Atrih, A.; Foster, S. J.; Buttner, M. J. *J. Bacteriol*. **1999**, *181*, 204–211. doi:10.1128/JB.181.1.204-211.1999
11. Chang, S.-C.; Yang, W.-C.; Lee, Y. H.; *Biochim. Biophys. Acta*. **1992**, *1129*, 219–222.

doi:10.1016/0167-4781(92)90491-h

12. Gomez-Escribano, J. P.; Bibb, M. J. *Methods Enzymol*. **2012**, *517*, 279–300. doi: 10.1016/B978-0-12-404634-4.00014-0

13. Ikeda, H.; Kazuo, S. Y.; Omura, S. *J Ind Microbiol Biotechnol*. **2014**, *41*, 233-250. doi:10.1007/s10295-013-1327-x

14. Bierman, M.; Logan, R.; O'Brien, K.; Seno, E. T.; Rao, R. N.; Schoner, B. E. *Gene*. **1992**, *116*, 43–49. doi:10.1016/0378-1119(92)90627-2
